# Clinical description, genetic analysis and characterization of two NLRP12 heterozygous VUS variants

**DOI:** 10.3389/fimmu.2026.1755711

**Published:** 2026-04-22

**Authors:** Asier Lizama-Muñoz, Juan Francisco Gutiérrez-Bautista, José María García-Aznar, Inmaculada Perea, Mónica Bernal, Miguel Ángel López-Nevot

**Affiliations:** 1 Department of Biochemistry, Molecular Biology and Immunology III, Faculty of Medicine, University of Granada, Granada, Spain; 2 Clinical Analysis and Immunology Department, University Hospital Virgen de las Nieves, Granada, Spain; 3 Institute for Biosanitary Research of Granada (ibs.GRANADA), Granada, Spain; 4 Programa de Doctorado en Biomedicina, University of Granada, Granada, Spain; 5 Health in Code S.L., A Coruña, Spain

**Keywords:** autoinflammatory disease, human inborn errors of immunity, molecular characterization, NLRP12 mutation, novel variation, phenotype and genotype, SAIDs

## Abstract

**Background:**

Systemic autoinflammatory diseases (SAIDs) are a rare, heterogeneous group of disorders caused by dysregulated innate immune activation.

**Objective:**

To characterize the phenotype and clinical features of two unrelated cases with SAID suspicion, each carrying a heterozygous *NLRP12* variant of uncertain significance (VUS)—one previously reported and one newly identified in Europeans—and to evaluate their functional and genetic relevance.

**Methods:**

We analyzed clinical and genetic findings from two individuals harboring distinct heterozygous *NLRP12* variants not associated with Familial Cold Autoinflammatory Syndrome type 2 (FCAS2). Functional assays using peripheral blood mononuclear cells (PBMCs) were performed to assess the impact of these variants on cytokine production. Clinical features were compared among affected and unaffected family members and correlated with molecular findings.

**Results:**

Genetic screening revealed two different single nucleotide variants (SNVs) in *NLRP12* gene. The first, c.2854G>A (p.Asp952Asn), previously reported but still classified as a VUS, substitutes a negatively charged aspartic acid with an uncharged asparagine at residue 952. Structural modeling suggested disruption of local interactions between the pyrin domain (PYD) and leucine-rich repeat (LRR) domains, potentially impairing protein activation. The second variant, c.616C>G (p.Arg206Gly), represents a novel finding in Europeans, replacing a large positively charged arginine with a small, flexible glycine at residue 206. Modeling predicted destabilization of a loop near the nucleotide oligomerization core domain (NACHT) and loss of key interactions. Despite their classification as VUS according to the American College of Medical Genetics and Genomics (ACMG) criteria, both variants demonstrated functional impact. Carriers of p.Asp952Asn (index case, mother, and sibling) showed a marked, generalized increase in proinflammatory cytokines, particularly IL-1β, TNF-α, and IL-17A. The index case exhibited exceptionally high basal IL-1β levels, consistent with an activated inflammatory state, and clinical data confirmed elevated C-reactive protein (CRP) levels. In contrast, the individual carrying p.Arg206Gly displayed a selective inflammatory response characterized by increases in IL-1β and TNF-α only after lipopolysaccharide (LPS) stimulation, not after muramyl dipeptide (MDP), with no elevation in IL-10 or IL-17A.

**Conclusion:**

Functional data support that both *NLRP12* variants affect inflammatory cytokine production, though with distinct patterns: p.Asp952Asn induces broad hyperinflammation, whereas p.Arg206Gly results in stimulus-specific dysregulation. These findings highlight the importance of integrating genetic, structural, and functional evidence for the interpretation of *NLRP12* VUS in the context of Human Inborn Errors of Immunity and primary immunodeficiencies.

## Introduction

Systemic autoinflammatory diseases (SAIDs), or autoinflammatory syndromes, are a rare, low-frequency, and heterogeneous group of disorders. They typically result from abnormal innate immune activity in the context of an underlying genetic variant (1, 2). Both their origin and clinical course are distinctive. Their main characteristic is the induction of a persistent inflammatory state without the production of antibodies or specific lymphocytes (1). This occurs because they affect various components of innate immunity, including macrophages, monocytes, neutrophils, and dendritic cells (DCs) (1–5). Another hallmark of these diseases is their episodic presentation, with inflammatory flares characterized by elevated systemic cytokine production, alternating with symptom-free intervals in which cytokine activity is absent (6).

More than 30 genes have been associated with this broad group of disorders, including *NOD2*, *NLRC4*, *NLRP12*, *NLRP1*, and *MEFV* (5–7). They can be classified according to the affected pathway or functional mechanism. For example, NLRC4 and NLRP1 alter the IL-18 production pathway; NOD2 modifies nuclear factor κB (NF-κB) function and dysregulates tumor necrosis factor (TNF) synthesis; and MEFV and NLRP12 influence inflammasome activation (3, 5, 7).

NLRP12-associated autoinflammatory disease (NLRP12-AID) commonly manifests with non-infectious periodic fever, elevated acute-phase reactants such as C-reactive protein (CRP) and erythrocyte sedimentation rate (ESR), urticaria or skin rash, headache, abdominal pain, and systemic inflammation, particularly affecting the skin, joints, muscles, and nerves. NLRP12-AID is usually caused by autosomal dominant (AD) mutations in the *NLRP12* gene (8–10).

**Figure 1:**
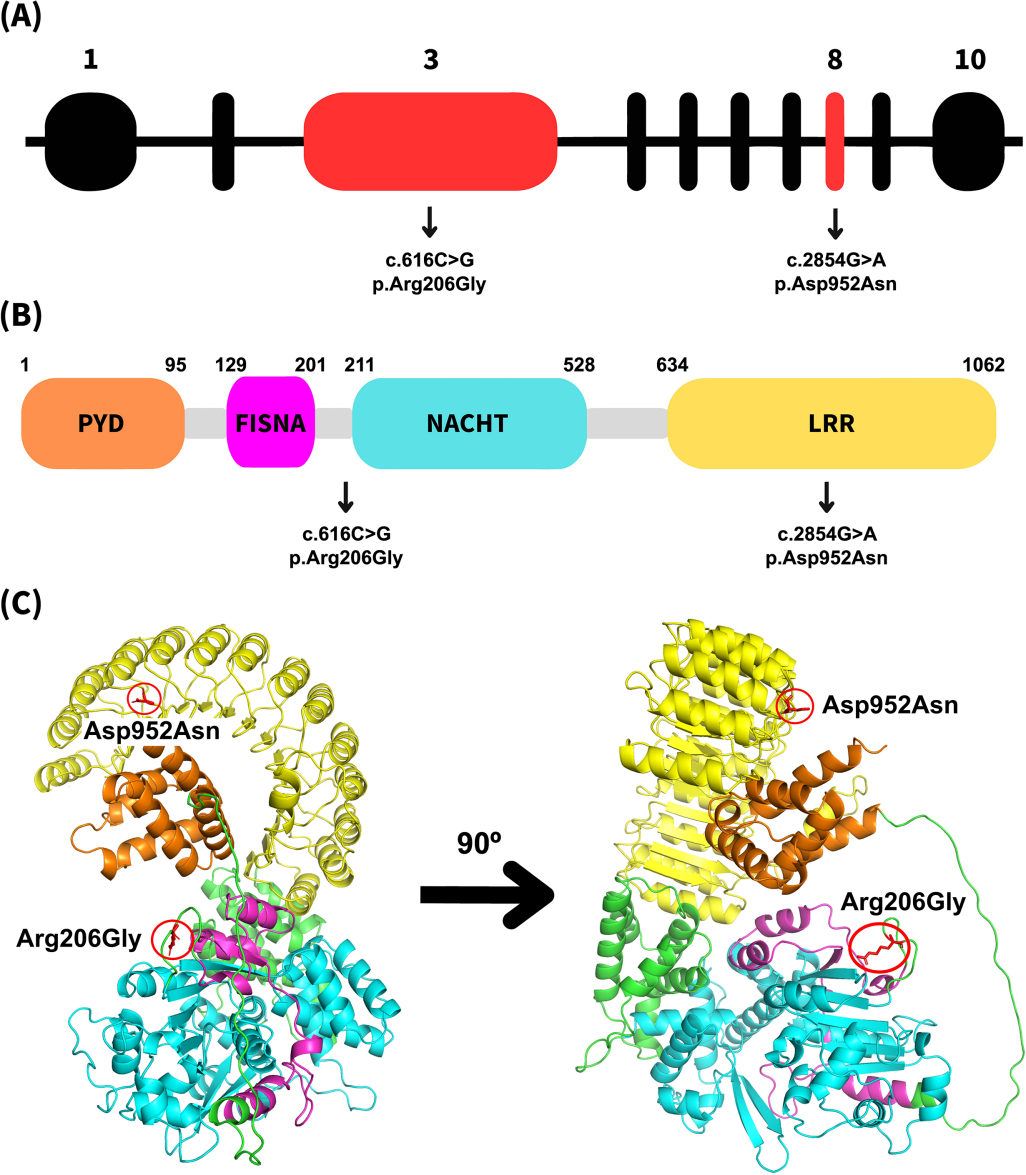
Schematic and structural representation of NLRP12. **(A)** Genomic structure of the human NLRP12 gene, highlighting exons 3 and 8 in red, which contain the variants identified in this study. **(B)** Schematic representation of the NLRP12 protein, showing the PYD domain in orange, the FISNA domain in magenta, the NACHT domain in cyan, and the LRR domain in yellow. The locations of the reported variants are indicated. **(C)** Ribbon diagram of the NLRP12 protein generated using PyMOL based on the AlphaFold-predicted structure AF-P59046-F1, highlighting the positions of both variants identified in this study. Domains are colored as in panel **(B)**, with interdomain regions shown in green.

The *NLRP12* gene, also known as *Monarch-1*, is located on chromosome 19q13.42 (11) and consists of 10 exons encoding a 1060-amino-acid protein (**Figure 1A**). It is an intracellular protein mainly expressed in innate immunity system cells such as neutrophils, eosinophils, macrophages and DCs (12–14). It belongs to the Nucleotide oligomerization domain (NOD)-like receptor (NLR) protein family and the NLRP subfamily. These proteins are characterized by a pyrin domain (PYD), a nucleotide-binding oligomerization domain (NBD), and a leucine-rich repeat (LRR) region (15–18). NLRP12 contains a single PYD domain (aa 1–95) at its N-terminus, a fish-specific nucleotide oligomerization core domain (NACHT)-associated domain (FISNA, aa 129–201), a nucleotide oligomerization core domain (NACHT, aa 211–528), and an LRR region (aa 634–1062) at its C-terminus (**Figures 1B, C**) (12, 19, 20).

**Figure 2:**
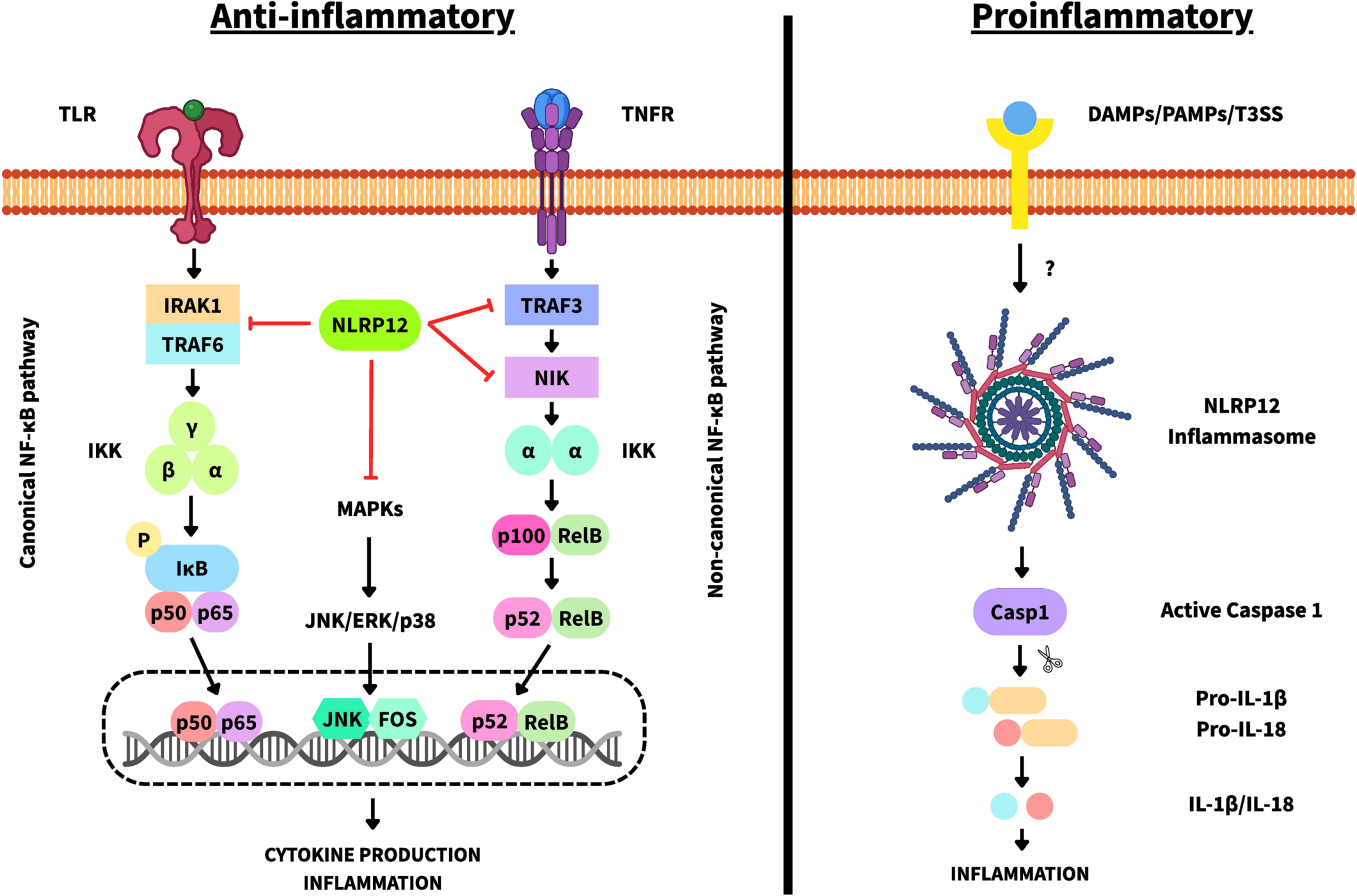
Schematic representation of NLRP12 functions. On the left, the anti-inflammatory roles of NLRP12 are summarized, highlighting the key signaling proteins and pathways in which NLRP12 has been shown to exert inhibitory activity. NLRP12 interacts with IRAK1 and TRAF6 to block TLR-dependent downstream signaling and the canonical NF-κB pathway; with TRAF3 and NIK to inhibit TNFR signaling and the non-canonical NF-κB pathway; and it can also modulate the mitogen-activated protein kinase (MAPK) pathway. On the right, the proinflammatory functions of NLRP12 are illustrated, emphasizing inflammasome assembly and its downstream effects. Upon activation by specific DAMPs, PAMPs, or type III secretion systems, NLRP12 undergoes oligomerization leading to inflammasome formation. This, in turn, activates caspase-1 (Casp1), resulting in the processing and release of the active forms of IL-1β and IL-18, thereby promoting inflammation. IκB: inhibitor of NF-κB; IKK: IκB kinase complex; JNK: c-Jun N-terminal kinase; ERK: extracellular signal-regulated kinase.

NLRP12 protein is thought to have a dual role. On the one hand, it acts as a negative regulator of inflammation by inhibiting both the canonical and non-canonical NF-κB pathways and caspase-1 (Casp1) activation, ultimately reducing the production of proinflammatory cytokines (**Figure 2**) (8, 13, 21–24). On the other hand, in response to certain pathogen-associated molecular patterns (PAMPs) or damage-associated molecular patterns (DAMPs)—which are not yet well defined—it can oligomerize and directly interact with Casp1, forming an inflammasome-like complex that produces the active forms of proinflammatory cytokines such as IL-1β and IL-18 (**Figure 2**) (25–28). It also exerts immunomodulatory effects on Toll-like receptors (TLRs) and other signaling pathways by directly interacting with and blocking intermediates such as interleukin-1 receptor-associated kinase 1 (IRAK1), TNF receptor-associated factor 6 (TRAF6), TNF receptor-associated factor 3 (TRAF3), and NF-κB-inducing kinase (NIK) (**Figure 2**) (8, 13, 21, 22). In addition, it has been reported to interact with NLRP3 and acts as a negative regulator of inflammasome assembly (29).

**Figure 3:**
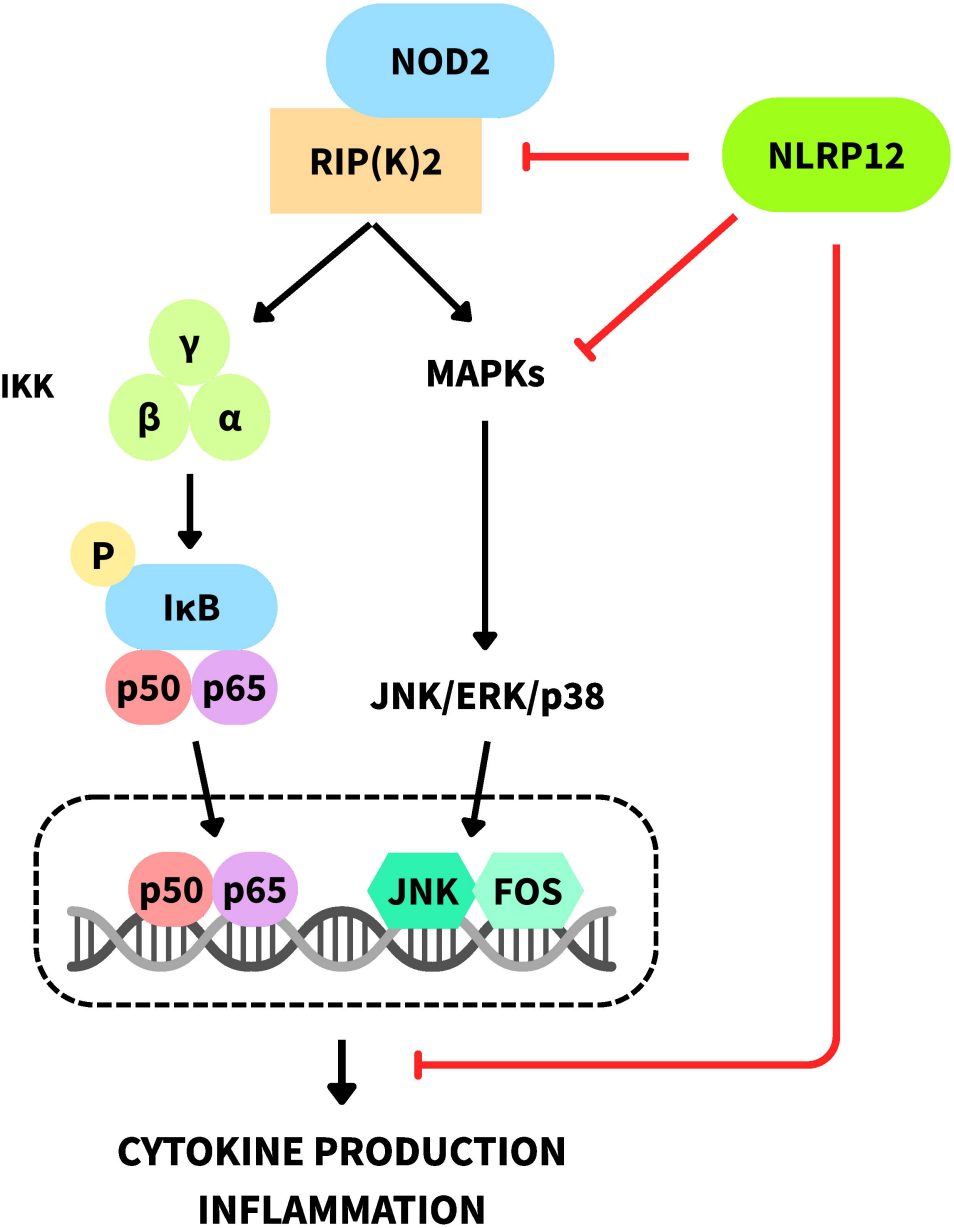
Schematic representation of NLRP12–NOD2 cross-regulation. Activation of RIP(K)2 by NOD2 promotes the activation of the IκB kinase (IKK) complex, leading to phosphorylation of IκB and release of NF-κB for nuclear translocation. In parallel, NOD2 signaling also activates the MAPK pathway. NLRP12 negatively regulates RIP(K)2-mediated activation of both the NF-κB and MAPK pathways triggered by NOD2. The inhibitory points of NLRP12 along the NOD2 pathway are highlighted in the figure. RIP2/RIPK2 is denoted as RIP(K)2. JNK: c-Jun N-terminal kinase; ERK: extracellular signal-regulated kinase.

Recent evidence indicates cross-regulation between NOD2 and NLRP12. NOD2 activation by muramyl dipeptide (MDP) induces its homo-oligomerization and interaction with RIP2 or RIPK2, subsequently activating the canonical NF-κB and MAPK-dependent pathways. NLRP12 can prevent activation of these pathways, thereby reducing proinflammatory cytokine production resulting from NOD2 activation (**Figure 3**) (30–33). Furthermore, NLRP12 has been shown to promote NOD2 degradation via the proteasome by hijacking HSP90, enhancing its immunoregulatory capacity. This immunosuppressive function plays an important role in gut microbiome colonization and tolerance (21, 30, 34).

Certain *NLRP12* gene variants have been associated with Familial Cold Autoinflammatory Syndrome type 2 (FCAS2, OMIM #611762) (5, 35). This syndrome results in recurrent systemic inflammation triggered by exposure to cold. It typically presents with an autosomal dominant inheritance pattern, reduced penetrance, and variable expressivity. To date, approximately ten mutations have been reported, most of which affect exon 3, considered a hotspot region. Dysregulated secretion of proinflammatory cytokines, such as IL-1β, caused by impaired inhibition of the NF-κB pathway has been linked to the disease (8, 36–38).

The most frequently identified *NLRP12* genetic alterations associated with SAIDs or NLRP12-AID are nonsense mutations, although many variants remain classified as “variants of uncertain significance” (VUS) due to insufficient functional or clinical evidence. In this study, we investigated the clinical manifestations and genetic background of two families carrying two different heterozygous *NLRP12* variants—one previously reported and classified as a VUS, and another novel variant in Europeans—not associated with FCAS2 but compatible with a SAID phenotype.

## Materials and methods

### Patients and controls

We evaluated the clinical cases of two patients with suspected SAID. When possible, additional close family members—including siblings and parents—were included to investigate inheritance patterns and identify potential genetic variants associated with SAIDs, provided they were willing to participate. We also include a group of controls in the study to compare functional assays results between cases and controls. All included individuals were of Caucasian ancestry, and the parents of each index case were non-consanguineous. The participants included in the study are summarized in **Table 1**.

**Table 1:** Demographic data of the participants included in the study.

Group	Case	Family member	Age (years)	Sex	Clinical information
Controls (n = 12)	–	–	F: 43.33 ± 14.68 M: 43.17 ± 10.30	F: 6 M: 6	Healthy controls
Cases	Family 1	Patient 1	32	F	Recurrent fever episodes of unspecified origin
	Family 1	Sibling	26	F	Asymptomatic carrier
	Family 1	Mother	57	F	History of fibromyalgia, arthralgia, hearing loss, and vertigo
	Family 1	Father	59	M	No relevant clinical information
	Case 2	Patient 2	51	M	Recurrent oral aphthae with fever and a VEXAS suspicion

F, Female; M, Male; VEXAS, Vacuoles; E1 enzyme, X-linked, autoinflammatory, somatic syndrome. Age is presented as mean ± standard deviation (SD) and sex distribution is presented as a number for controls and case participants are reported individually.

Peripheral blood samples were obtained from all available participants. Total genomic DNA was extracted from peripheral blood mononuclear cells (PBMCs) for genetic analysis, and PBMCs were also used for functional assays. Samples were obtained during a stable period for both analyses.

This study involving human participants was reviewed and approved by the Portal de Ética de la Investigación Biomédica, Junta de Andalucía (Protocol Codes: 0297-N-21 and TFM-EEAI-2024). Written informed consent was obtained from all participants prior to inclusion.

### Molecular analysis

Genomic DNA was analyzed using next-generation sequencing (NGS) with Twist Bioscience kits for molecular amplification and library preparation. Sequencing was performed on the Illumina NextSeq 1000 platform, and data analysis and variant interpretation were carried out using SOPHiA DDM™ for Genomics. Sequence alignment was performed against the GRCh37/hg19 human reference genome. All identified variants of interest were subsequently validated by targeted Sanger sequencing.

The customized gene panels included the coding regions and flanking intronic sequences of 575 genes associated with primary immunodeficiencies or human inborn errors of immunity, as previously described (5). These panels also encompassed all SAID-related genes, including *MEFV, NLRC4, NLRP1, NLRP12, NLRP3*, and *NOD2*. The complete gene panel used in this study is provided in the Supplementary Materials (**Supplementary Table 1**).

### Gene frequency

Allele frequencies of the identified gene variants were obtained from publicly available population databases, specifically the Genome Aggregation Database (gnomAD) (39) and the Allele Frequency Aggregator (ALFA) database (40).

### Evaluation of variants pathogenicity

Genomic data were analyzed using the NCBI reference transcript NM_144687. The potential impact of the identified variants on protein function was assessed using a comprehensive set of in silico predictive tools, including PolyPhen-2 (41), MutationT@ster2 (42), MutationTaster 2021 (43), SIFT (44), MutationAssessor (45), FATHMM (46), REVEL (47), MetaRNN (48), BayesDel (49), GERP (50), CADD (51), DANN (52), AlphaMissense (53), GenoCanyon (54), and fitCons (55). We also evaluated the consequences of the amino acid substitutions using ProtVar (56), and interpreted the results using the Franklin data analysis platform (Genoox, Tel Aviv, Israel) (57) and the SpliceAI toolkit (58, 59). The outputs generated by these algorithms are summarized in **Supplementary Tables 2**, **3**. Variant interpretation was further guided by the American College of Medical Genetics and Genomics (ACMG) classification criteria (60). To explore the structural impact of the variants, in silico molecular modeling and structural analyses of the NOD2 protein were performed. A three-dimensional representation was used to assess how the amino acid substitutions could affect local structural environments and functional domains. PyMOL (version 2.5.7, Schrödinger, LLC) (61) was used for molecular visualization. Structural models of the variants were generated using MODELLER (version 10.6) (62, 63) with default parameters. The native structure of NLRP12was modeled based on the AlphaFold-predicted structure (AF-P59046-F1) (64, 65). All structural graphics presented in this article were created using PyMOL based on the models described above (61).

### Human PBMC isolation

Peripheral blood from each participant, as well as from healthy individuals, was collected using EDTA-coated Vacutainer^®^ tubes. Human PBMCs were then isolated by a Ficoll-Paque density gradient technique as previously described, with minor modifications (66–71). Ficoll gradient centrifugation was performed at 2000 rpm for 20 min at room temperature (RT), followed by two wash steps consisting of centrifugation at 1000 rpm for 10 min each. After the final wash, the resulting cell pellet was resuspended in 2 mL of fresh culture medium—minimal essential medium (MEM) supplemented with glutamine, 1% fetal bovine serum (FBS), and 1% antibiotic mix (gentamicin/vancomycin). An aliquot of approximately 150 μL was taken for cell counting.

Cell counts were obtained using the Sysmex XN-1000™ automated hematology analyzer. Immediately after isolation, PBMCs were used for cell culture experiments.

### Cell culture and stimulation

Cells were seeded at a density of 1 × 10^6^ cells per mL in each well of a 96-well flat-bottom plate and maintained in a 5% CO_2_ incubator at 37 °C for 24 or 48 hours following stimulation (66–68, 71, 72).

Because NLRP12 protein lacks a specific known stimulant, PBMC activation was performed by stimulating cells with 500 ng/mL lipopolysaccharide from Escherichia coli O111:B4 (LPS; LPS25-1MG, Sigma-Aldrich) and 1 μg/mL N-acetylmuramyl-L-alanyl-D-isoglutamine hydrate, also known as muramyl dipeptide (MDP; A9519-1MG, Sigma-Aldrich), according to previously published protocols (26–28, 34, 36–38, 73). Cell culture supernatants were collected 24 or 48 hours after stimulation and stored at −80 °C until analysis.

### Cytokine analysis

IL-1β, IL-4, IL-6, IL-8, IL-10, IL-12(p70), IL-17A, IL-18, and TNF-α in cell culture supernatants were quantified using a magnetic bead–based multiplex assay with commercially available custom Mix & Match ProcartaPlex™ Immunoassay kits (Thermo Fisher Scientific), following the manufacturer’s instructions. Plates were read using the Luminex™ FLEXMAP 3D™ system with xPONENT^®^ software (ThermoFisher).

### Complementary blood tests

Additional blood tests were performed on whole blood samples collected from each participant to further assess their overall health status.

These analyses included the evaluation of key cellular and systemic parameters, with a particular focus on immune system composition and activity. Specifically, we measured C-reactive protein (CRP), complement components, immunoglobulin levels, and immune cell subpopulations (**Supplementary Figure 1**; **Supplementary Table 4**).

### Statistics

Cytokine concentrations (pg/mL) were obtained using a 5-parameter logistic (5PL) weighted regression calculated by the xPONENT^®^ software integrated into the Luminex™ FLEXMAP 3D™ system. Each measurement was performed in duplicate.

GraphPad Prism v8.0.2 was used for data analysis and for generating the graphical representations included in this study. Results were displayed as box plots, with individual participant values overlaid on each plot.

Due to experimental constraints and sample availability, each participant was evaluated only once; therefore, no statistical significance testing was performed. Instead, individual values were compared with those of control samples using Z-scores for absolute values (Equation 1) and for relative values normalized to basal production (Equation 2). Values exceeding ±3 standard deviations (SD) from the control mean were considered outside the normal range. All data used can be consulted in Supplementary Section, specifically are summarized in **Supplementary Table 6–10**.

(1) Z = (x−μ)/σ

Where x represents the cytokine value under analysis, μ represents the mean cytokine level in controls, and σ represents the standard deviation of cytokine levels in controls.

(2) ΔZ = (Δx−Δμ)/Δσ

Where Δx (Δx = stimulated condition − basal production) represents the relative increase in the cytokine under analysis, Δμ represents the mean relative increase in controls, and Δσ represents the standard deviation of the relative increase in control samples.

## Results

### Clinical features

For the first family, we evaluated a patient (Patient 1) who experienced several episodes of fever of unspecified origin (**Table 1**) in her 30’s. The age of onset of the current SAID was when she was 30 years. The first documented episode occurred in August 2023, when she presented with fever and a rash on the thigh. After a few days, the symptoms resolved, leaving only residual marks from the rash. In December 2023, she experienced another severe febrile episode without an identifiable cause. This episode presented with chills but no rash, and the symptoms resolved after one week. Throughout 2024, she experienced additional similar episodes, with a particularly notable event in May, during which she exhibited the highest CRP peak recorded (**Supplementary Figure 1**; **Supplementary Table 4**). She also presented with a febrile episode in September 2024, accompanied by fever and asthenia, which resolved within a few days. In 2025, she experienced further CRP peaks associated with fever, although these were lower than the episode in May 2024.

She additionally reported a history of headaches and recurrent diarrhea since childhood, oral aphthae beginning with the onset of fever episodes, and lateral cervical lymphadenopathy. Metabolic disorders and bacterial infections were ruled out as potential causes of the fever. Her clinical presentation was consistent with a nonspecific SAID based on the signs and symptoms described. Treatment included paracetamol or ibuprofen to manage fever episodes, and omeprazole as gastric protection. Complementary treatments were administered when necessary. Her mother reported a history of fibromyalgia, arthralgia, and hypoacusis with vertigo episodes. No other relevant symptoms or clinical manifestations suggestive of autoinflammatory diseases were identified in the family history.

The second index case (Patient 2) was referred due to suspected Behçet’s disease, mouth and genital ulcers with inflamed cartilage (MAGIC) syndrome, or vacuoles, E1 enzyme, X-linked, autoinflammatory, somatic syndrome (VEXAS) (**Table 1**). The current disease age of onset was when he was 45 years. The patient reported a history of recurrent oral aphthae and joint pain accompanied by fever for several years. The most recent episode, one year prior, lasted two weeks and involved painful oral aphthae that impaired swallowing, painful genital ulcers, skin lesions (erythema nodosum, periorbital inflammation, auricular chondritis), cervical lymphadenopathy, fever of nonspecific origin, and arthralgia. Histopathology biopsied from an oedematous plaque of the first toe on the right foot revealed vasculitic changes consistent with Behçet’s disease. He also presented with hypoacusis and had experienced recent weight loss due to the severity of oral aphthae. He reported that his mother also suffered from recurrent oral aphthae.

Complementary blood tests revealed a reduction in nearly all immune cell subsets. Total CD45+ cells were decreased by approximately 6% (1499 per µL; normal range: 1600–2400). T cells were reduced, with CD3+ lymphocytes decreased by about 14% (830 per µL; normal range: 960–2600), primarily due to a 40% reduction in CD3+CD4+ lymphocytes (327 per µL; normal range: 540–1660). CD3+CD8+ lymphocytes remained within the normal range (429 per µL; normal range: 270–930). NK cells (CD3–CD16+CD56+) were increased by approximately 17% (597 per µL; normal range: 127–509). B cells were decreased, with CD19+ cells reduced by about 46% (66 per µL; normal range: 122–632). IgE levels were substantially elevated, with an increase of about 40% (137 IU/mL; normal range: 26–100), while total IgG was slightly reduced by approximately 15% (471 mg/dL; normal range: 570–1822). Complement components and CRP were within normal limits. The clinical presentation was compatible with a SAID and a Behçet-like phenotype, although the underlying cause remained unknown.

### Genetic analysis

To investigate whether a genetic background could underlie the suspicion of SAID, molecular analysis of genomic DNA was performed. A single nucleotide variant (SNV) in the *NLRP12* gene was identified in each case: one in Patient 1 and a different one in Patient 2.

In Patient 1, the identified variant rs140792345–c.2854G>A (p.Asp952Asn) results in the substitution of aspartic acid by asparagine at residue 952, located in exon 8 within the LRR domain (**Figure 1**). This substitution replaces a polar, hydrophilic, negatively charged amino acid with a polar, hydrophilic but uncharged residue, a change that may alter internal protein interactions.

In Patient 2, the variant c.616C>G (p.Arg206Gly) leads to the substitution of arginine by glycine at residue 206, located in exon 3 between the FISNA and NACHT domains, where no disease-causing variants have previously been described (8) (**Figure 1**). This substitution replaces a polar, hydrophilic, positively charged residue with a small, non-polar, hydrophobic, and flexible amino acid, potentially altering local structural conformation and interactions.

The p.Asp952Asn variant has been previously reported by LabCorp Genetics – Invitae (San Francisco, California, United States) in 2019 and by the Genome Diagnostics Laboratory – Hospital for Sick Children (Toronto, Ontario, Canada) in 2022 (74, 75). It is listed as a variant of uncertain significance (VUS) in public databases (76, 77).

In contrast, the p.Arg206Gly variant occurs at a residue in which other substitutions have been described—rs111754022–c.616C>T/A (p.Arg206Cys/Ser)—reported by Atschekzei et al. (2022) and Yun et al. (2023) (78, 79), both considered VUS (76, 80, 81). To our knowledge, the arginine-to-glycine substitution has not been previously reported in the literature or clinical variant databases.

To further assess segregation and exclude a *de novo* mutation, targeted Sanger sequencing was performed in close family members—including parents and siblings—when possible (**Table 2**). This was feasible only for the first family; the second family declined participation. Thus, for Patient 2 only the index case genotype was available.

**Table 2:** Genetic analysis results of the genomic DNA sequencing.

Family member	Locus	Nucleotide variation	Amino acid variation	Domain affected	Carried	ClinVar classification
Patient 1	Chr 19q13.42	c.2854G>A	p.Asp952Asn	Exon 8 - LRR	Heterozygous	Uncertain significance
Sibling of patient 1	Chr 19q13.42	c.2854G>A	p.Asp952Asn	Exon 8 - LRR	Heterozygous (asymptomatic carrier)	Uncertain significance
Mother of patient 1	Chr 19q13.42	c.2854G>A	p.Asp952Asn	Exon 8 - LRR	Heterozygous	Uncertain significance
Father of patient 1	Chr 19q13.42	c.2854G>A	p.Asp952Asn	Exon 8 - LRR	Not carried	Uncertain significance
Patient 2	Chr 19q13.42	c.616C>G	p.Arg206Gly	Exon 3 - NACHT	Heterozygous	Not included

Chr, chromosome; LRR, leucine-rich repeat region; NACHT, nucleotide oligomerization core domain.

**Figure 4:**
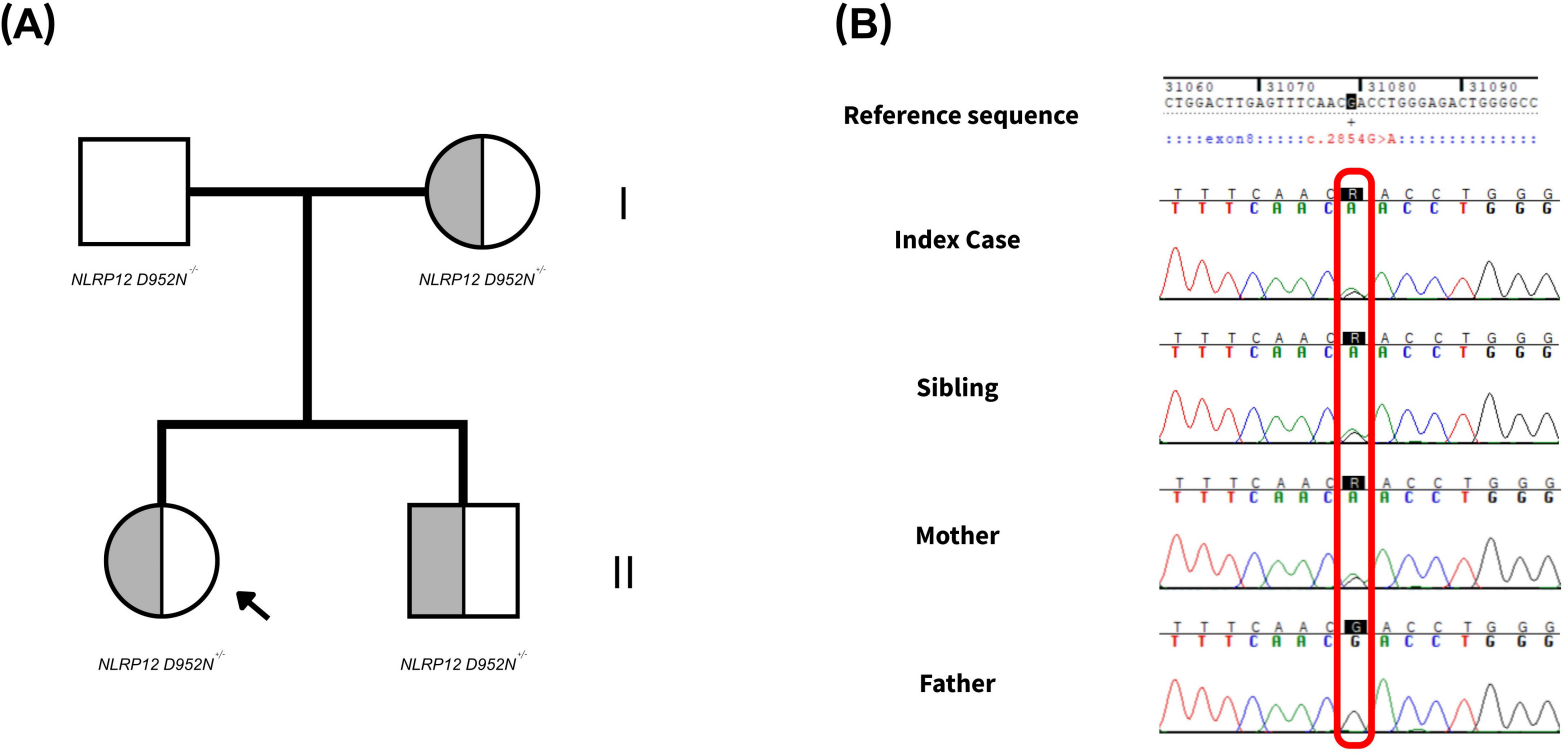
Sequencing outcomes of the family under study. **(A)** Pedigree of the family analyzed in this study. Each symbol is divided into two halves representing the two NLRP12 alleles. The left half of each symbol indicates the presence of the p.Asp952Asn variant, while the right half represents the wild-type allele. Shading denotes heterozygosity for the variant. The arrow marks the index case in whom the variant was first identified. **(B)** Sanger sequencing results for the c.2854G>A (p.Asp952Asn) variant. The position of the substitution is highlighted. Full Sanger chromatograms for the family are provided in **Supplementary Figure 1**.

In the first family, genetic analysis revealed that both the mother and sibling of Patient 1 carried the same variant identified in the index case, whereas the father did not (**Table 2**). These findings support maternal transmission of the variant, which is present in heterozygosis in all carriers. The results are shown in **Figure 4**, and full Sanger chromatograms are provided in the Supplementary Material (**Supplementary Figure 2**).

For Patient 2, genetic analysis confirmed that he did not carry any variants associated with Behçet’s disease, MAGIC syndrome, or VEXAS, and specifically no mutation in the UBA1 gene. Instead, he carried the heterozygous NLRP12 VUS described above, for which little information is currently available (**Table 2**).

### Differential diagnosis

For both cases, during differential diagnosis carried out only a SAID was considered as the possible disease, due to the atypical presentation of the symptoms and the diverse phenotypes founded. Periodic Fever, Aphthous Stomatitis, Pharyngitis, and Adenitis (PFAPA) and other similar autoinflammatory diseases were discarded by clinicians because patients don’t meet all typical symptoms and the molecular analysis carried out did not reveal any related genes. Two uncertain significance variants in *NLRP12* gene were founded. In addition, PFAPA usually affect children, but in this case, we have two clinical cases with ages of onset in adulthood (30 and 45 for Patient 1 and Patient 2, respectively). For all above, an atypical NLRP12-related SAID was considered as the current disease.

### Evaluation of the pathogenicity

According to the multiple bioinformatic prediction tools used, both variants were predominantly classified as likely pathogenic or deleterious, based on their predicted impact on protein structure and function resulting from the amino acid substitutions (**Supplementary Tables 1**, **2**). However, following the ACMG criteria (29), both variants are currently classified as VUS, as each meets only one moderate-level criterion (PM2: absence or rarity in population databases).

We next evaluated allele frequencies using data from gnomAD (39) and ALFA (40). For the p.Asp952Asn variant, the reported allele frequency in the general population was 0.001425% and 0.0005085% in the European (non-Finnish) population according to gnomAD (39, 82, 83), with no homozygous individuals reported (**Table 3**). In the ALFA database, frequencies were even higher—0.013% in the general population—but the variant has not been reported in Europeans (40, 83). Based on these data, the p.Asp952Asn variant should be considered rare (allele frequency < 0.01).

**Table 3:** Mutation frequencies of NLRP12 p.Asp952Asn and p.Arg206Gly variants in all data available on GnomAD and ALFA for the general population (total) and for the European (non-Finnish) and European population respectively, where Spanish population is included.

Variant information	Variant	Total - GnomAD	European (non-Finnish) - GnomAD	Total - ALFA	European - ALFA
Allele Count	p.Asp952Asn	23	6	4	0
	p.Arg206Gly	8	0	N/A	N/A
Allele Number	p.Asp952Asn	1614028	1180026	30412	19780
	p.Arg206Gly	1612576	1178774	N/A	N/A
Allele Frequency	p.Asp952Asn	1.425e-5	5.085e-6	1.3e-4	0.00
	p.Arg206Gly	4.961e-6	0.00	N/A	N/A

N/A, not available.

For the p.Arg206Gly variant, information was available only in gnomAD (39, 84), where its allele frequency in the general population was 0.0004964%, with no carriers identified in Europeans. This supports its classification as novel within the European population (**Table 3**). According to these data, this variant also meets the criteria to be considered rare (allele frequency < 0.01).

To further assess the structural and functional implications of these mutations, we performed in silico structural modeling using MODELLER and visualized the resulting models with PyMOL.

**Figure 5:**
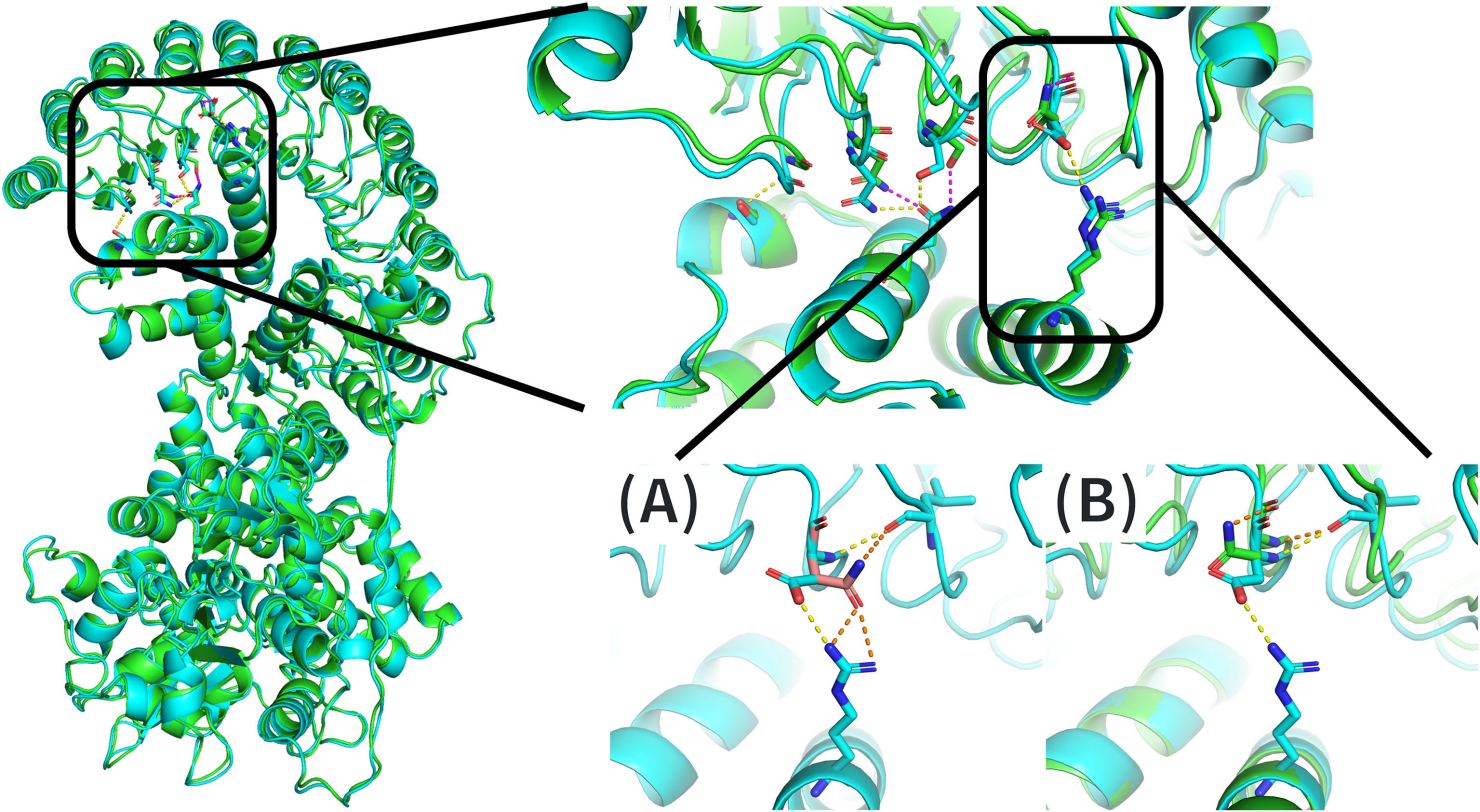
Three-dimensional modeling of residue 952 using PyMOL. The left panel shows the full structure of the NLRP12 protein, with the AlphaFold-predicted structure depicted in blue and the MODELLER-generated mutant structure in green. The upper right image shows a magnified view of the affected region, highlighting the predicted interactions between the PYD and LRR domains; colors correspond to those in the left panel. The lower panel presents two comparative visualizations of the possible structural consequences of the amino acid substitution at residue 952. **(A)** The native residue, aspartic acid (blue), is shown together with its expected interacting neighboring residues (also in blue), with predicted hydrogen-bond interactions highlighted in yellow. The predicted mutant residue (pink) is superimposed, and potential new interactions are shown in orange. **(B)** Comparison between the AlphaFold-predicted structure (blue) and the MODELLER-generated mutant (green), showing the hypothetical native interactions in yellow and predicted new interactions in orange.

For the p.Asp952Asn substitution, the replacement of a negatively charged amino acid with an uncharged residue that may disrupt the local environment by altering charge distribution and intermolecular interactions. The change from a carboxyl group to an amine group appears to modify the interaction between the LRR and PYD domains in the closed state. This was based on comparation between the predicted native structure (AlphaFold prediction) with our modelled mutant structure (**Figure 5**). In the closed conformation, an interaction seems to exist between the LRR and PYD domains that helps to stabilize the protein. This stabilization appears to be mediated by interactions between Asp952 and Arg7; Ser979, Asn1007 and Gln58; and Gly1036 and Ser47 (**Figure 5**).

The amino acid substitution at residue 952 could alter the interaction between residue 952 and Arg7, potentially strengthening it (**Figure 5A**), which would be associated with a higher energy requirement for NLRP12 activation. However, the MODELLER prediction suggested the opposite: the substitution at residue 952 may result in loss of the interaction with Arg7, favouring a new intramolecular interaction instead (**Figure 5B**). According to ProtVar predictions, this amino acid position is moderately conserved, and protein stability (based on predicted ΔΔG values) is not affected (**Supplementary Table 2**).

**Figure 6:**
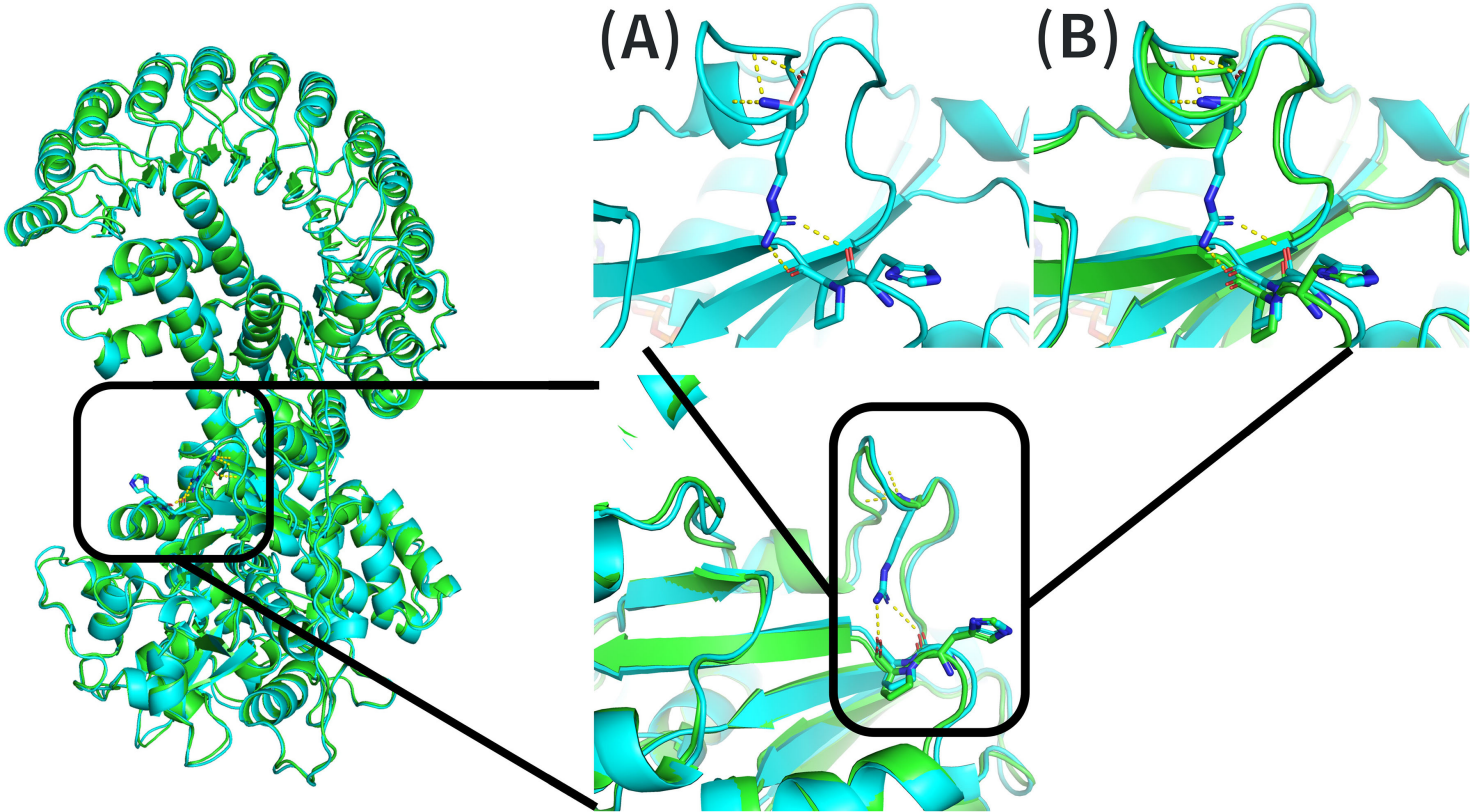
Three-dimensional modeling of residue 206 using PyMOL. The left panel shows the full structure of the NLRP12 protein, with the AlphaFold-predicted structure displayed in blue and the MODELLER-generated mutant structure in green. The lower right panel shows a magnified view of the affected region, using the same color scheme. The upper panel illustrates two comparative predicted views of the structural consequences of the amino acid substitution at residue 206: **(A)** The native residue, arginine (blue), together with its expected interacting neighboring residues (also in blue), with predicted hydrogen-bond interactions highlighted in yellow. The predicted mutant residue, glycine, is superimposed in pink. **(B)** Comparison between the AlphaFold-predicted structure (blue) and the MODELLER-generated mutant (green), showing the hypothetical native interactions in yellow.

For the p.Arg206Gly variant, the substitution of a large, polar, hydrophilic, positively charged residue with a small, non-polar, hydrophobic, and flexible amino acid could cause the destabilization of a local loop structure. Modeling indicated that in the predicted native protein structure, Arg206 could interact with His356 and Pro357. These interactions may be lost with the amino acid change at position 206. Comparison of native and mutant models revealed a possible structural shift in which these interactions disappear, allowing the loop to open and potentially altering the protein structure near the NACHT domain (**Figure 6**). According to ProtVar predictions, conservation at this position is relatively low, and protein stability is likely affected based on predicted ΔΔG values (**Supplementary Table 3**).

### Functional assays

To evaluate the functional impact of the identified variants, we performed functional assays based on PBMC stimulation and cytokine production analysis.

#### IL-1β production

**Figure 7:**
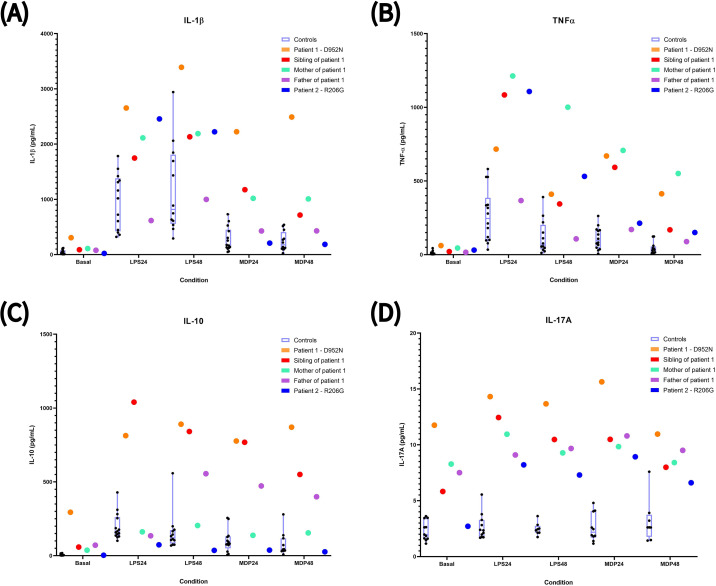
Production of different cytokines determined in peripheral blood mononuclear cells (PBMCs) in different conditions. The data are shown as individual points for each sample and the whisker plot for the control group represents the median (centre line), interquartile range (box) and standard deviation (whiskers). The cytokines levels were measured from PBMCs culture supernatant from various individuals: healthy controls (black dots in light blue boxes), patient 1 with the D952N mutation (orange dots), her sibling (red dots), her mother (green dots) and her father (purple dots), as well as the patient 2, with carries the R206G mutation (blue dots). Cells were cultivated for 24 or 48 hours under different conditions: basal (cell culture without any stimulant), LPS (cell culture with lipopolysaccharide as stimulant) and, MDP (cell culture stimulated with muramyl dipeptide). Each point was determined twice. Pannel **(A)** represents the results obtained from IL-1β. Pannel **(B)** contains result from TNFα. Pannel **(C)** represents the results from the IL-10. And pannel **(D)**, the values obtained from IL-17A.

For Patient 1 (p.Asp952Asn), IL-1β production was extraordinarily high under all conditions—both at baseline and after stimulation with LPS or MDP (**Figure 7**). This was reflected in Z-scores exceeding 3 standard deviations (SD) above the controls in virtually every experimental setting, for both absolute and relative production, indicating strong inflammatory activation (**Table 4**).

**Table 4:** Z-score results for each experimental condition and for absolute and relative production of the IL-1β, TNFα, IL-10 and IL-17A.

*Cytokine*	*Condition*	*Patient 1 (p.Asp952Asn)*	*Sibling of patient 1*	*Mother of patient 1*	*Father of patient 1*	*Patient 2 (p.Arg206Gly)*
IL-1β	Basal	**6.49**	1.08	1.62	0.84	-0.56
	LPS24	**3.35**	1.56	2.28	-0.67	*2.96*
	LPS48	*2.72*	1.17	1.24	-0.22	1.28
	MDP24	**8.97**	**4.16**	**3.43**	0.73	-0.29
	MDP48	**12.74**	*2.70*	**4.36**	1.08	-0.30
	ΔLPS24	*2.89*	1.51	2.20	-0.75	**3.07**
	ΔLPS48	2.41	1.12	1.17	-0.27	1.32
	ΔMDP24	**10.10**	**5.21**	**4.16**	0.88	-0.09
	ΔMDP48	**13.25**	*2.88*	**4.68**	1.03	-0.22
TNF-α	Basal	**4.37**	0.85	*2.95*	0.44	1.73
	LPS24	2.49	**4.52**	**5.23**	0.56	**4.65**
	LPS48	2.49	1.92	**7.56**	-0.11	**3.53**
	MDP24	**7.51**	**6.48**	**8.02**	0.81	1.38
	MDP48	**9.36**	**3.13**	**12.87**	1.10	*2.68*
	ΔLPS24	2.36	**4.76**	**5.37**	0.58	**4.83**
	ΔLPS48	2.06	1.84	**7.37**	-0.19	**3.39**
	ΔMDP24	**6.59**	**6.10**	**7.31**	0.56	0.93
	ΔMDP48	**8.35**	*2.90*	**12.46**	0.90	2.15
IL-10	Basal	**58.87**	**10.83**	**6.52**	**13.42**	-0.45
	LPS24	**6.79**	**9.28**	-0.38	-0.68	-1.35
	LPS48	**5.52**	**5.15**	0.34	*2.99*	-0.93
	MDP24	**8.70**	**8.59**	0.48	**4.78**	-0.81
	MDP48	**10.17**	**6.07**	1.01	**4.14**	-0.64
	ΔLPS24	**3.18**	**7.89**	-0.84	-1.46	-1.39
	ΔLPS48	**3.19**	**4.56**	0.03	2.36	-0.96
	ΔMDP24	**5.73**	**9.02**	0.22	**4.56**	-0.74
	ΔMDP48	**6.51**	**5.42**	0.58	**3.30**	-0.64
IL-17A	Basal	**10.65**	**3.96**	**6.73**	**5.86**	0.45
	LPS24	**9.98**	**8.38**	**7.09**	**5.50**	**4.74**
	LPS48	**19.87**	**14.20**	**12.08**	**12.78**	**8.57**
	MDP24	**10.28**	**6.21**	**5.70**	**6.46**	**4.99**
	MDP48	**3.97**	2.45	*2.66*	**3.22**	1.74
	ΔLPS24	*2.88*	**7.72**	**3.02**	1.72	**6.38**
	ΔLPS48	2.29	**6.07**	1.04	*2.65*	**6.00**
	ΔMDP24	**3.45**	**4.14**	1.37	*2.92*	**5.54**
	ΔMDP48	-1.25	0.86	-0.59	0.73	2.08

Z-scores above 3 standard deviations (SD) are highlighted in bold with a shaded box, and values close to 3 SD (greater than 2.5 SD) are highlighted in italics.

The sibling and mother of Patient 1 (p.Asp952Asn) showed a similar cytokine production pattern, particularly after MDP stimulation (**Figure 7**). Their Z-scores confirmed this, exceeding 3 SD only in the MDP-stimulated conditions (**Table 4**).

In contrast, the father—who does not carry the variant—displayed cytokine levels comparable to controls, with Z-scores close to 0 and within the −1 to 1 range (**Figure 7**; **Table 4**).

For Patient 2 (p.Arg206Gly), IL-1β production showed a completely different behavior. Elevated levels were observed only after LPS stimulation (**Figure 7**). Z-scores confirmed this pattern: absolute production at 24 hours approached 3 SD, and relative production exceeded 3 SD (**Table 4**).

#### TNF-α production

A similar tendency was observed for TNF-α. Patient 1 (p.Asp952Asn) showed markedly elevated TNF-α levels under most experimental conditions, particularly after MDP stimulation (**Figure 7**). Z-scores exceeded 3 SD in both basal and MDP-stimulated conditions (**Table 4**).

The sibling and mother of Patient 1 (p.Asp952Asn) also showed elevated cytokine levels across conditions, with the mother displaying the highest production (**Figure 7**). Their Z-scores were above 3 SD in most conditions, both for absolute and relative production, with the mother showing especially pronounced values (**Table 4**).

As observed for IL-1β, the father showed no significant differences from controls, with an almost identical cytokine profile and unremarkable Z-scores (**Figure 7**; **Table 4**).

For Patient 2 (p.Arg206Gly), TNF-α levels increased markedly only after LPS stimulation (**Figure 7**). Z-scores exceeded 3 SD exclusively under LPS-stimulated conditions, both for absolute and relative production (**Table 4**).

#### IL-10 production

For Patient 1 (p.Asp952Asn), IL-10 production showed a strong tendency toward increased secretion, especially at baseline (**Figure 7**). Z-scores exceeded 3 SD in all experimental conditions for both absolute and relative values. Basal IL-10 was exceptionally high—approximately 60 SD above the control mean (**Table 4**).

The sibling of Patient 1 (p.Asp952Asn) showed a similar IL-10 profile, with Z-scores above 3 SD in all conditions, whereas the mother showed only a slight increase at baseline, with Z-scores close to 0 (**Figure 7**; **Table 4**).

Interestingly, the father showed a noticeable increase in IL-10 secretion (**Figure 7**). Z-scores exceeded 3 SD in many conditions, for both absolute and relative production (**Table 4**).

For Patient 2 (p.Arg206Gly), IL-10 production did not increase, showing a pattern similar to controls (**Figure 7**). All Z-scores were below 3 SD, typically with negative values, indicating a downward trend (**Table 4**).

#### IL-17A production

For Patient 1 (p.Asp952Asn), IL-17A production was the highest among all subjects (**Figure 7**). Z-scores exceeded 3 SD in all experimental conditions, with basal levels exceeding 10 SD. Relative increases were also substantial, reflecting strong baseline and stimulated responses (**Table 4**).

The sibling and mother of Patient 1 (p.Asp952Asn) showed the same pattern but with lower cytokine levels (**Figure 7**). Their Z-scores were above 3 SD for absolute production in most conditions but not consistently for basal levels, suggesting that stimulation induced production without an exaggerated baseline response (**Table 4**).

The father, although a non-carrier, showed a slight rise in IL-17A, with Z-scores > 3 SD particularly after stimulation (**Figure 7**; **Table 4**).

For Patient 2 (p.Arg206Gly), IL-17A levels were elevated under nearly all experimental conditions except baseline (**Figure 7**). Z-scores exceeded 3 SD for absolute and relative production after LPS stimulation and, notably, also after MDP stimulation at 24 hours (**Table 4**).

#### Other cytokines

The remaining cytokines showed no meaningful differences from controls or produced inconsistent findings; thus, these data are not included.

#### Overall interpretation

The markedly elevated production of IL-1β, TNF-α and IL-17A in Patient 1 (p.Asp952Asn) and her mother suggests that both were likely experiencing a disease flare at the time of sampling. This interpretation is supported by elevated inflammatory markers such as CRP in the index case (but not the mother) (**Supplementary Figure 1**; **Table 4**). Combined, these findings strongly support active autoinflammatory disease in the index case during testing.

In summary, the c.2854G>A (p.Asp952Asn) variant is associated with consistently increased production of pro-inflammatory cytokines (especially IL-1β and TNF-α) across all carriers.

In contrast, the c.616C>G (p.Arg206Gly) variant identified in Patient 2 induces increased IL-1β and TNF-α only in response to LPS, while MDP responses resemble those of controls (**Figure 7**).

## Discussion

SAIDs constitute a very low-frequency and heterogeneous group of Human Inborn Errors of Immunity (HIEI) or primary immunodeficiencies caused by abnormal activity of the innate immune system. They may arise from variants in more than 30 genes, including *NOD2, NLRC4, NLRP12, NLRP1*, and *MEFV*, among others (2–5). To date, only a small number of pathogenic or likely pathogenic mutations in *NLRP12* have been reported, supported by clear clinical or functional evidence. In contrast, many additional variants remain classified as variants of uncertain significance (VUS) according to ClinVar (76, 77).

In this study, we identified two different *NLRP12* variants in heterozygosity: a novel mutation in Europeans, c.616C>G (p.Arg206Gly), not previously reported, and a previously described variant, c.2854G>A (p.Asp952Asn), also classified as a VUS. We described the genotype and characteristics of a family carrying the p.Asp952Asn variant and a second unrelated patient carrying the p.Arg206Gly variant. Both variants were associated with a loss of function (LoF) of NLRP12, resulting in increased release of proinflammatory cytokines. Other *NLRP12* mutations have been associated with rare autosomal dominant SAIDs (NLRP12-AID) or FCAS2, in which a weakened inhibition of NF-κB and an upregulation of inflammatory cytokines have been described (8, 85). NLRP12-AID, like other SAIDs, is typically presented during childhood but recently, there are an increasing number of reported cases where the presentation of the SAID is on adulthood (3, 86, 87).

Despite functional validation strategies using site-directed mutagenesis in heterologous systems such as HEK293T cells can provide additional mechanistic insight, the protein under study, NLRP12, exhibits cell-type–restricted expression and is predominantly expressed in innate immune cells (12–14). Therefore, epithelial cell lines such as HEK293T may not reproduce the physiological signaling context required for NLRP12 function. Furthermore, inflammasome sensors operate within complex multiprotein signaling platforms whose activity is highly dependent on the cellular environment, and overexpression-based systems may introduce non-physiological signaling artifacts (13, 14, 22, 88–90). Importantly, previous studies support a mechanistic role for NLRP12 in the regulation of inflammatory responses. NLRP12 has been shown to modulate canonical and non-canonical NF-κB signaling pathways as well as MAPK-dependent inflammatory signaling. Experimental models further demonstrate that loss or down-regulation of NLRP12 enhances inflammatory responses, while certain patient-derived mutations promote spontaneous caspase-1 activation (13, 22, 23, 27, 91, 92). Together, these findings support the biological plausibility that alterations in NLRP12 function may directly contribute to hyperinflammatory phenotypes and for that reason, we decided to follow a different strategy that combine the using of its naturally expression tissue by using PBMCs obtained from controls and patients to simulate the cellular environment and the stimulation with LPS and MDP similar to similar previous works (26–28, 34, 36–38, 93).

Although cytokine levels can fluctuate during disease activity, functional ex-vivo stimulation assays using PBMCs are widely employed to investigate innate immune dysregulation in autoinflammatory diseases, particularly in rare autoinflammatory diseases such as FMF, CAPS or CRMO (94–96). In these conditions, cells after LPS stimulation frequently exhibit exaggerated cytokine responses –particularly IL-1β, IL-18 or IL-6– and can discriminate autoinflammatory diseases and reflect disease activity at the time of evaluation. Several studies have employed and validated that cytokine secretion measured in cell culture supernatants after stimulation produces a disease-specific inflammatory signatures and can even assist in the differential diagnosis of autoinflammatory syndromes. These assays therefore provide a robust functional readout of inflammasome activity even in cross-sectional experimental settings (93–98). For that reason, single-point ex vivo cytokine assays reliably identified the inflammatory phenotype in affected family members, validating our procedure employed.

The distinct functional behavior of the two variants can be explained by their different domain locations in NLRP12. Members of the NLR family share a conserved architecture consisting of a central NACHT domain with ATPase activity, and a C-terminal LRR region (8, 13, 21–24). The NACHT domain plays a key role in nucleotide binding and conformational switching, which are essential steps in inflammasome activity and downstream signaling. Variants placed in this domain or located near it may therefore directly affect receptor activation dynamics and downstream signaling (79, 99–103). In contrast, the C-terminal LRR region is primarily involved in ligand sensing and maintenance of the autoinhibited conformation of the receptor through intramolecular interactions. Alterations in this region are more likely to influence receptor regulation or sensitivity to stimulation and activation rather than the conformational switching (79, 99–101, 104–106). Similar domain-dependent functional effects have been reported for other NLR family members such as NLRP3 or NOD2, supporting the notion that variants located near the NACHT domain and in the C-terminal region may produce distinct functional outcomes (79, 99, 100). Based on it, we propose that the p.Asp952Asn variant impairs proper activation of the protein by enhancing structural interactions between the LRR and PYD domains in the closed conformation -inactive state-. This may prevent the protein from performing its immunomodulatory function, leading to a LoF phenotype. For the p.Arg206Gly variant, we propose that the substitution affects the region between the FISNA and NACHT domains without disrupting the NBD, that results in a barrier for conformational switching and downstream dynamics alteration. As a result, this variant may cause a partial LoF, altering specific protein interactions.

NLRP12 interacts with several intermediates of inflammatory pathways, including RIPK2 (RIP2), IRAK1, TRAF6, NLRP3, and HSP70/90, modulating their proinflammatory activity (13, 21, 29, 34, 107–112). For the p.Asp952Asn variant, we hypothesize that the altered protein conformation prevents or weakens these interactions, impairing regulation of the canonical NF-κB pathway and enhancing inflammasome formation, ultimately increasing IL-1β and TNF-α production (8, 13, 21, 22, 25–28).

Upon LPS stimulation, NLRP12 normally modulates the TLR4 downstream signaling pathway by interacting with intermediates such as IRAK1 or TRAF6. The p.Asp952Asn variant would impede this regulation, enabling excessive cytokine production and NLRP3 inflammasome activation (13, 29, 111–115). Conversely, after MDP stimulation, NLRP12 should hijack HSP70/90 and interact with RIPK2 to limit NOD2 signaling; however, the variant would prevent this interaction, stabilizing NOD2 and allowing exaggerated NF-κB activation, inflammasome formation, and cytokine production (21, 34, 72, 109, 110, 116–119). Because NOD2 activation is generally weaker than TLR4 activation, cytokine levels were consistently higher after LPS stimulation than after MDP stimulation. Our results are coherent with those from Li et al. (2023), who identified similar patterns in patients with related NLRP12 variants (26).

The observed clinical spectrum within the family—ranging from severe autoinflammatory symptoms to an asymptomatic state—is a well-documented phenomenon in SAIDs and consistent with the incomplete penetrance observed in other NLRP12-AIDs and variable expressivity frequently reported in NLRP12-associated disorders (9, 15, 79, 101, 120–123). Rather than weakening the evidence for pathogenicity, this variability suggests a ‘multi-hit’ model of disease manifestation. Our data suggests that while the variant lowers the biochemical barrier for NF-κB activation, the genotype alone is insufficient to dictate the clinical outcome. Instead of this, the final phenotype is determined by a complex interplay between the primary genetic defect (the variation founded in *NLRP12*) and a wider signaling network, influenced by sex-bias, microbiome and epigenetics.

A critical factor is the observed female-restricted phenotypic affectation that may be produced by the interaction between NLRP12 and X-linked regulatory networks. Key mediators of the innate immune response—including TLR7, IRAK1, and the NF-κB essential modulator (NEMO/IKBKG)—are encoded on the X-chromosome (124–127). We hypothesize that the skewed X-chromosome inactivation (XCI) or the escape from inactivation of these loci may create a pro-inflammatory cellular environment. This suggested model produces an increased “dosage” or activity of X-linked signaling components that act as a synergistic driver exacerbating the loss of NLRP12-mediated regulation (128–132). Consequently, this can explain all observed phenotypes, from severe SAID to the asymptomatic carrier. In the asymptomatic and the mild carriers may have a more “regulatory” X-linked profile. The mild symptoms observed in the older carrier suggest that age-related hormonal stabilization that may dampen the hyper-inflammatory response (133–135).

The severe phenotype also requires epigenetic reprogramming of innate immune cells and/or microbiome alterations. Trained immunity involves the induction of an ‘inflammatory memory’ following exposure to specific stimuli, leading to a hyper-responsive state upon subsequent challenges (136, 137). Microbiome and trained immunity influence in autoinflammatory and autoimmune diseases presentation has been widely investigated (137–146). In this context, we hypothesize that in the severely affected patient, the loss of NLRP12 inhibition in NF-κB pathway synergizes with the pro-inflammatory state produced by trained immunity and the microbiome dysregulation for disease debuting. For all above, these observations indicate that the NLRP12 c.2854G>A variant is not an isolated driver, but it acts as a biochemical primer that requires a specific biological and epigenetic context to manifest a SAID, as occur in other autoinflammatory disorders (147).

For the p.Arg206Gly variant, we hypothesize that NLRP12 retains its ability to regulate the NOD2 pathway. Thus, it can hijack HSP70/90 and promote proteasome-dependent NOD2 degradation (116, 148, 149), as well as interact with RIPK2, enabling proper control of MDP-induced signaling (21, 34, 72, 109, 110, 116–119). In contrast, its ability to block TLR4-dependent signaling through IRAK1 or TRAF6 appears to be impaired (13, 29, 111–115), resulting in the observed increased cytokine production—particularly IL-1β and TNF-α—after LPS stimulation. IRAK1 is especially relevant in this context because has been demonstrated that NNLRP12 interacts with it through its NACHT domain (111), and this interaction lost may explain why only LPS stimulation produced a marked response. In addition, we observed pronounced IL-17A production independent of the stimulus, consistent with Behçet’s or Behçet-like disease, where IL-17A elevation has been frequently reported (150–152).

In all cases, the observed increase in IL-10 production among individuals who also exhibited elevated IL-1β and TNF-α levels may reflect compensatory activation of anti-inflammatory mechanisms in response to the strong proinflammatory stimulation *in vitro*. This phenomenon has been described previously, consistent with the literature (153–160).

Elevation in cytokine responses follow by innate immune stimulation is well documented and reflects the inter-individual variability in innate immune responsiveness among healthy individuals. Ex vivo stimulation assays using PBMCs following by LPS or MDP stimulation have demonstrated the existence of different types of responses among healthy individuals (161–164). Particularly, IL-10 represents a key regulatory cytokine that is physiologically induced following activation of pattern recognition receptors (PRR) such as TLR4 or NOD2, acting as a feedback mechanism to limit excessive inflammation. Multiple immune cell populations present in PBMC cultures—including monocytes, dendritic cells, and lymphocytes—can contribute to IL-10 production upon stimulation (164–166). Moreover, IL-17A production can vary considerably depending on the inflammatory stimuli and the cellular composition of the culture. In addition to Th17 cells, IL-17A can also be produced by other innate-like lymphocyte populations (167–169). For that reason, inter-individual variability in relative abundance of the PBMCs or activation state of these cell populations may lead to variable cytokine outputs. Therefore, the increased IL-10 and IL-17A levels observed in a non-carrier (the father) likely reflect normal biological variability in innate immune responsiveness, and also some of the controls have a slightly elevated response.

A notable finding in our study is the discrepancy between computational predictors and experimental data. While bioinformatic predictions are useful for variant prioritization, they remain insufficient to establish their pathogenicity. Functional assays therefore represent a critical step to validate the biological impact of candidate variants, particularly in the context of Inborn Errors of Immunity (170–173). In these particular types of disease mechanisms often involve complex regulatory or signalling defects that may not be captured by computational models. High-throughput algorithms often rely heavily on evolutionary conservation and may fail to capture gain-of-function or regulatory-loss mechanisms (174–177).

The missense variant *NLRP12* c.2854G>A (p.Asp952Asn) is a rare genetic variation discovered in a heterozygous state in a Spanish family. While some bioinformatic algorithms such as REVEL and AlphaMissense classified this variant as benign, our functional analysis demonstrated a marked increase in pro-inflammatory cytokine production after stimulation with LPS and MDP. This hyper-inflammatory response supports that the substitution leads to a functional impairment of NLRP12, likely through a decreased ability to suppress the canonical and non-canonical NF-κB pathway (8, 13, 21–24), validating its pathogenic potential, regardless of the in-silico scores predictions. This functional evidence supports the application of the ACMG PS3 criterion (as strong). The observed clinical spectrum within the family (from severe SAID to an asymptomatic carrier) is consistent with the incomplete penetrance and variable expressivity frequently reported in other NLRP12-associated disorders (15, 79, 120, 121). This backs the meeting of the PP1 and PP4 criterion as supporting state. Due to its extreme rarity in population databases (gnomAD - 0.0005085% and ALFA also reports low frequencies) (39, 40, 82, 83), the experimental evidence of immune dysregulation, and the clinical segregation, we propose the reclassification of the *NLRP12* c.2854G>A variant from a “Variant of Uncertain Significance (VUS)” to a “Likely Pathogenic”, according to the fulfilling ACMG guidelines criteria PS3, PM2, PP1, and PP4. The detailed information of the strength applied and the explanation for considering the meeting criteria is shown in **Table 5**.

**Table 5:** Applied ACMG/AMP criteria for reclassification of the *NLRP12* c.2854G>A and *NLRP12* c.616C>G variants based on functional evidence.

Variant	Criterion	Strength	Explanation for its meeting
NLRP12 c.2854G>A (p.Asp952Asn)	PS3	Strong	*In vitro* functional studies demonstrate a significant increase in pro-inflammatory cytokine production (IL-1β, TNF-α) after LPS and MDP stimulation, consistent with a loss of inhibitory control in the NF-κB pathway
	PM2	Moderate	The variant is extremely rare in population databases - GnomAD (1.425e-5 in total and 5.085e-6 in Europeans), which is below the threshold for autoinflammatory diseases – It had already met this criterion
	PP1	Supporting	The variant co-segregates with the disease phenotype within the family across multiple affected individuals, including asymptomatic
	PP4	Supporting	The patients’ clinical presentation is highly specific for a SAID; a phenotype strongly associated with NLRP12 dysfunction
NLRP12 c.616C>G (p.Arg206Gly)	PS3	Moderate	*In vitro* assays prove a significant increase in pro-inflammatory cytokine release (LPS-induced) compared to wild-type (controls)
	PM2	Moderate	The variant is extremely rare in the GnomAD population database (4.961e-6 for total population and new in Europeans) and not included in ALFA, which is below the threshold for autoinflammatory diseases – It had already met this criterion as Support state

LPS, lipopolysaccharide; MDP, muramyl dipeptide; SAID, Systemic Autoinflammatory Disease.

The other missense variant identified in *NLRP12* gene, the c.616C>G (p.Arg206Gly) is also a rare genetic finding identified also in a heterozygous state. Even several in silico meta-predictors (such as REVEL and BayesDel) suggest a neutral effect, the variant is absent in the European (non-Finnish) population -our case represents the first description of this variant in heterozygosity in Europeans- and shows an extremely low global frequency in gnomAD (39, 40, 82, 83), fulfilling the PM2 criterion as moderate state. In contrast, functional characterization provides evidence for its pathogenicity: the variant leads to a significant increase in pro-inflammatory cytokine production following by LPS stimulation. This dysregulation of the NLRP12 anti-inflammatory response is a hallmark of NLRP12-AIDs (15, 79, 120, 121), justifying the application of the ACMG PS3 criterion. Although the functional evidence is not as strong as for the p.Asp952Asn variant, we suggest only its application in a moderate state. Given the correlation between the experimental functional impact and the clinical findings, the weight of the biological evidence and population rarity outweighs the conflicting computational predictors used. For that reason, we propose the reclassification of the *NLRP12* c.616C>G variant from a “VUS variant” to “Likely Pathogenic”, according to the ACMG guidelines meeting criteria PS3 and PM2. The detailed information on its strength and the explanation for considering its meeting is reflected in **Table 5**.

## Conclusion

Based on our findings, we identified and characterized two different NLRP12 variants in heterozygosity, each altering NLRP12 function in distinct ways. The p.Asp952Asn variant, previously reported but never in Europeans, demonstrated a stimulus-independent impact on cytokine production, leading to increased IL-1β, TNF-α, and IL-17A levels. In contrast, the p.Arg206Gly variant—reported here for the first time—showed a stimulus-dependent effect, with marked dysregulation after LPS but not MDP stimulation, suggesting a selective impairment of TLR4-dependent pathways. Both variants result in NLRP12 LoF and support the presence of autoinflammatory phenotypes distinct from FCAS2, further expanding the spectrum of NLRP12-associated diseases.

This study has limitations. First, the analysis is based on a single family and a single unrelated patient, limiting genotype–phenotype correlations. Studies involving larger cohorts are needed to confirm these associations. Second, we could not assess inheritance of the Arg206Gly variant because the family declined participation. Third, discrepancies among in silico tools and structural models highlight limitations of computational approaches, emphasizing the need for further experimental validation.

Despite these limitations, our work provides important insights into SAIDs and their underlying molecular mechanisms. By characterizing these previously unreported variants, we contribute valuable evidence that may support improved diagnostic interpretation, variant classification, and future therapeutic strategies in Human Inborn Errors of Immunity and primary immunodeficiencies.

## Data Availability

The data presented in the study are deposited in the National Center for Biotechnology Information (NCBI) repository, accessionnumber PZ290493.
