# Supplementary material for: Clinical description, genetic analysis and characterization of two NLRP12 heterozygous VUS variants
Source: Front Immunol. 2026 Apr 22;17:1755711. doi: 10.3389/fimmu.2026.1755711 (PMC13143751; doi:10.3389/fimmu.2026.1755711)
Supplement: Supplementary file 1 [file DataSheet1.docx]

**Supplementary Table 1**. Complete panel of 575 analysed genes related with Primary Immunodeficiencies (PID).

| **Gene panel used** |
| --- |
| *ABCB1, ACD, ACP5, ACTB, ADA, ADA2, ADAM17, ADAMTS13, ADAR, ADGRE2, AICDA, AIRE, AK2, ALPI, ANGPT1, ANXA11, AP1S3, AP3B1, AP3D1, APOL1, ARHGEF1, ARPC1B, ASAH1, ATAD3A, ATG16L1, ATG4A, ATM, ATP6AP1, B2M, BACH2, BANK1, BCL10, BCL11B, BLK, BLM, BLNK, BLOC1S6, BRCA1, BRCA2, BRIP1, BTK, BTNL2, C1QA, C1QB, C1QC, C1QTNF4, C1R, C1S, C2, C2orf69, C3, C4A, C4B, C5, C6, C7, C8A, C8B, C8G, C9, CALCOCO2, CARD11, CARD14, CARD9, CARMIL2, CASP10, CASP8, CCBE1, CCDC88B, CCL2, CCR1, CCR3, CCR5, CCR9, CD14, CD19, CD226,CD247, CD27, CD28, CD3D, CD3E, CD3G, CD4, CD40, CD40LG, CD46, CD55, CD59, CD70, CD79A, CD79B, CD81, CD8A, CDC42, CDCA7, CEBPE, CFB, CFD, CFH, CFHR1, CFHR2, CFHR3, CFHR4, CFHR5, CFI, CFP, CFTR, CHD7, CHUK, CIB1, CIITA, CLCN7, CLEC16A, CLEC7A, CLPB, COPA, COPG1, CORO1A, CR1, CR2, CRACR2A, CSF2RA, CSF2RB, CSF3R, CTC1, CTLA4, CTNNBL1, CTPS1, CTSC, CXCL13, CXCR2, CXCR4, CXCR5, CYBA, CYBB, CYBC1, DBR1, DCLRE1B, DCLRE1C, DDX58, DEF6, DGAT1, DGKE, DIAPH1, DKC1, DNAJC21, DNASE1, DNASE1L3, DNASE2, DNMT3A, DNMT3B, DOCK2, DOCK8, EFL1, EGFR, EIF2AK2, ELANE, ELF4, EPCAM, EPG5, ERAP1, ERBB2, ERBIN, ERCC4, ERCC6L2, ETS1, ETS2, EXTL3, F12, FAAP24, FADD, FANCA, FANCB, FANCC, FANCD2, FANCE, FANCF, FANCG, FANCI, FANCL, FANCM, FAS, FASLG, FAT4, FCGR2A, FCGR2B, FCGR3A, FCGR3B, FCHO1, FCN3, FERMT1, FERMT3, FNIP1, FOXN1, FOXP3, FPR1, FUT2, G6PC3, G6PD, GATA1, GATA2, GFI1, GINS1, GUCY2C, HAS2, HAVCR2, HAX1, HELLS, HMOX1, HNF1A, HS3ST6, HTRA2, HYOU1, ICAM1, ICOS, ICOSLG, IFIH1, IFNAR1, IFNAR2, IFNG, IFNGR1, IFNGR2, IGKC, IGLL1, IKBKB, IKBKG, IKZF1,I KZF2, IKZF3, IL10, IL10RA, IL10RB, IL12A, IL12B, IL12RB1, IL12RB2, IL17F, IL17RA, IL17RC, IL18BP, IL18R1, IL1RN, IL21, IL21R, IL23A, IL23R, IL2RA, IL2RB, IL2RG, IL36RN, IL6, IL6R, IL6ST, IL7R, INO80, IRAK1, IRAK4, IRF2BP2, IRF3, IRF4, IRF5, IRF7, IRF8, IRF9, IRGM, ISG15, ITCH, ITGAM, ITGB2, ITK, ITPKB, JAGN1, JAK1, JAK2, JAK3, KDM6A, KLRC4, KMT2A, KMT2D, KNG1, KRAS, LACC1, LAMTOR2, LAT, LCK, LCP2, LIG1, LIG4, LIMK2, LPIN2, LRBA, LRP5, LRRC8A, LSM11, LYST, MAD2L2, MAGT1MAL,* ***MEFV****, MMACHC, MOGS, MPO, MRC1, MRTFA, MS4A1, MSH6, MSN, MST1, MTHFD1, MVK, MYD88, MYH9, MYO5B, MYOF, MYSM1, NBAS, NBN, NCF1, NCF2, NCF4, NCKAP1L, NCSTN, NEIL1, NEUROG3, NFAT5, NFE2L2, NFKB1, NFKB2, NFKBIA, NHEJ1, NHP2,* ***NLRC4, NLRP1, NLRP12, NLRP3, NOD2****, NOP10, NOS2, NPM1, NRAS, NSMCE3, OAS1, ORAI1, OSTM1, OTULIN, PALB2, PARN, PAX1, PDCD1, PDGFRA, PEPD, PGM3, PHF11, PIK3CD, PIK3CG, PIK3R1, PLA2R1, PLCG2, PLEKHM1, PLG, PMS2, PNP, POLA1, POLD1, POLD2, POLE, POLE2, POLR3A, POLR3C, POLR3F, POMP, POU2AF1, PRF1, PRKCD, PRKDC, PROC, PRPS1, PSENEN, PSMA3, PSMB10, PSMB3, PSMB4, PSMB8, PSMB9, PSMG2, PSTPIP1, PTEN, PTPN2, PTPN22, PTPRC, RAB27A, RAC2, RAD51, RAD51C, RAG1, RAG2, RAI, RANBP2, RASGRP1, RBCK1, RC3H1, RECQL4, REL, RELA, RELB, RFWD3, RFX5, RFXANK, RFXAP, RHOG, RHOH, RIPK1, RMRP, RNASEH2A, RNASEH2B, RNASEH2C, RNF168, RNF31, RORC, RPSA, RTEL1, SAA1, SAMD9, SAMD9L, SAMHD1, SASH3, SBDS, SEC61A1, SEMA3E, SERPING1, SGPL1, SH2D1A, SH3BP2, SH3KBP1, SHOC2, SIAE, SKIV2L, SLC11A1, SLC26A3, SLC29A3, SLC35C1, SLC37A4, SLC39A7, SLC46A1, SLC7A7, SLC9A3, SLX4, SMARCAL1, SMARCD2, SNORA31, SNX10, SOCS1, SP110, SPATA5, SPI1, SPINK5, SPINT2, SPPL2A, SRP54, SRP72, STAT1, STAT2, STAT3, STAT4, STAT5B, STAT6, STIM1, STK4, STN1, STX11, STXBP2, SYK, TAP1, TAP2, TAPBP, TAZ, TBK1, TBX1, TBX21, TCF3, TCF7, TCIRG1, TCN2, TERT, TET2, TFRC, TGFB1, TGFBR1, TGFBR2, THBD, TICAM1, TINF2, TIRAP, TLR1, TLR2, TLR3, TLR5, TLR7, TLR8, TLR9, TMC6, TMC8, TMEM173, TNF, TNFAIP3, TNFRSF11A, TNFRSF13B, TNFRSF13C, TNFRSF1A, TNFRSF4, TNFRSF9, TNFSF11, TNFSF12, TNFSF13, TNFSF15, TNFSF4, TNIP1, TOM1, TOP2B, TP53, TPP2, TRAF1, TRAF2, TRAF3, TRAF3IP2, TREX1, TRIM21, TRIM22, TRNT1, TTC37, TTC7A, TYK2, UBA1, UBAC2, UBE2L3, UBE2T, UHRF1BP1, UNC119, UNC13D, UNC93B1, UNG, USB1, USP18, VPS13B, VPS45, VTN, WAS, WDR1, WIPF1, WRAP53, XIAP, XRCC2, ZAP70, ZBTB24, ZNF341, ZNFX1* |

Bold values indicate autoinflammatory syndromes-related genes.

**Supplementary Table 2**. Results of the predictive bioinformatic tools used for the c.2854G>A (p.D952N) variant classified by type of prediction and showing their obtained score and the interpretation of this value.

|  |  | ***NM_144687.4:c.2854G>A (p.D952N)*** | |
| --- | --- | --- | --- |
|  |  | ***Score*** | ***Interpretation*** |
| **Meta scores** | MetaLR | 0.19 | Likely benign |
|  | REVEL | 0.18 | Benign |
|  | BayesDel | -0.58 | Benign |
| **Individual Predictions** | GERP | 4.42 | Probably deleterious |
|  | Mutation assessor | 0.43 | Neutral |
|  | FATHMM | 0.68 | Uncertain |
|  | Mutation Taster | 1 | Deleterious |
|  | SIFT | 0.449 | Benign |
|  | PolyPhen2 | 0.949 | Probably damaging |
|  | CADD | 19.5 | Potentially deleterious |
|  | DANN | 0.99 | Deleterious |
|  | AlphaMissense | 0.16 | Benign |
| **Functional Whole Genome** | GenoCanyon | 0.01 | Benign |
|  | fitCons | 0.55 | Deleterious |
| **ProtVar** | Conservation | 0.47 | Moderate |
|  | Stability change ΔΔG | 0.71 | Unlikely to be destabilising |

**Supplementary Table 3**. Results of the predictive bioinformatic tools used for the c.616C>G (p.R206G) variant classified by type of prediction and showing their obtained score and the interpretation of this value.

|  |  | ***NM_144687.4:c.616C>G (p.R206G)*** | |
| --- | --- | --- | --- |
|  |  | ***Score*** | ***Interpretation*** |
| **Meta scores** | MetaLR | 0.45 | Likely benign |
|  | REVEL | 0.29 | Benign |
|  | BayesDel | -0.28 | Benign |
| **Individual Predictions** | GERP | 3.2 | Likely to be deleterious |
|  | Mutation assessor | 1.42 | Low deleterious probability |
|  | FATHMM | -0.81 | Uncertain |
|  | Mutation Taster | 0 | Benign |
|  | SIFT | 0.148 | Benign |
|  | PolyPhen2 | 0.944 | Probably damaging |
|  | CADD | 18.5 | Potentially deleterious |
|  | DANN | 0.97 | Deleterious |
|  | AlphaMissense | 0.21 | Benign |
| **Functional Whole Genome** | GenoCanyon | 0 | Benign |
|  | fitCons | 0.62 | Deleterious |
| **ProtVar** | Conservation | 0.42 | Fairly low |
|  | Stability change ΔΔG | 2.77 | Likely to be destabilising |

**Supplementary Table 4**. Results of the analysis of C-reactive protein (CRP) from 2024 until May 2025.

| ***Date of analysis*** | ***Value (<5 mg/L)*** |
| --- | --- |
| 11/01/2024 | **6,3** |
| 23/02/2024 | **11,18** |
| 22/04/2024 | 3,56 |
| 07/05/2024 | **43,49** |
| 28/06/2024 | 4,21 |
| 18/09/2024 | **6,5** |
| 25/09/2024 | 4,88 |
| 07/11/2024 | **5,2** |
| 10/01/2025 | **10,79** |
| 05/02/2025 | **7,45** |
| 16/04/2025 | 2,26 |

Bold values indicate results outside the normal range.

**Supplementary Figure 1**. Graphical representation of the C-reactive protein (CRP) values evaluated from 2024 until May 2025. Red line represents the threshold for normal CRP.


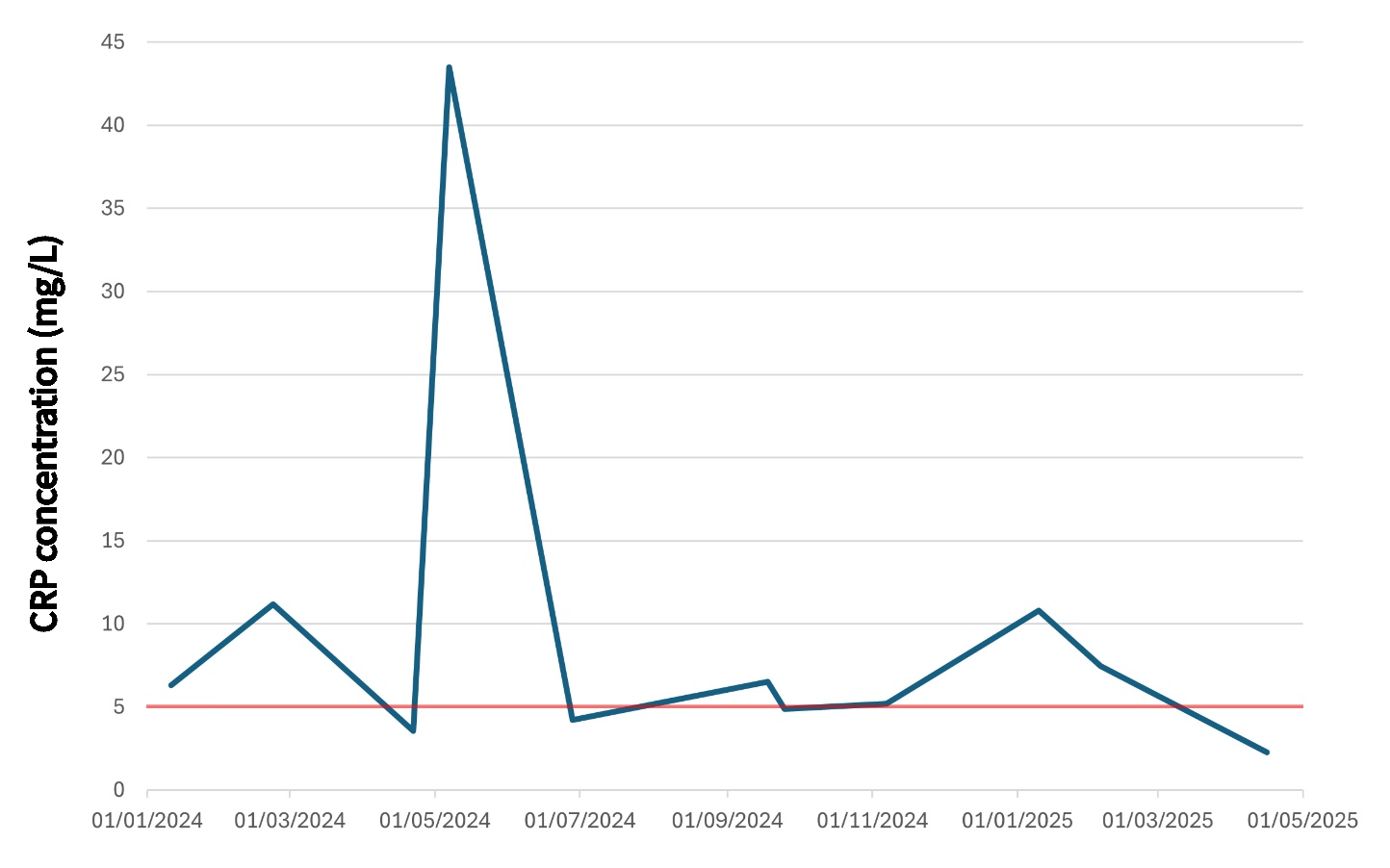


**Supplementary Figure 2**. Complete Sanger chromatographs results obtained from each patient where the forward and the reverse DNA was sequenced, and the result is compared with the reference sequence.


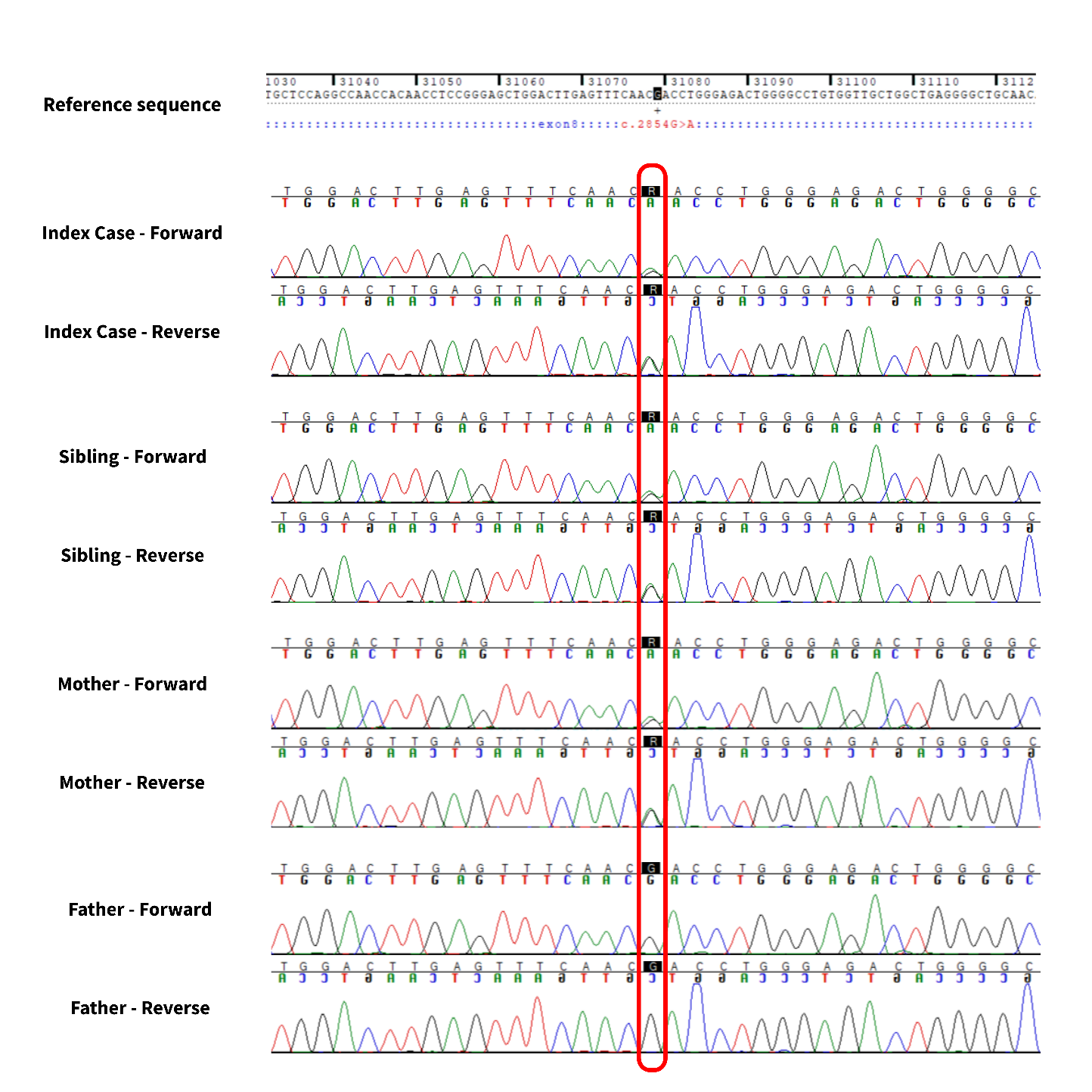


**Supplementary Table 5.** Row data used in statistical analysis (Z-scores) for control group

| ***Cytokine*** | ***Condition*** | ***Mean*** | ***SD*** | ***N*** |
| --- | --- | --- | --- | --- |
| **IL-1β** | Basal prod | 44,9647545 | 40,24932541 | 12 |
|  | LPS24 | 957,443135 | 506,9491615 | 12 |
|  | LPS48 | 1179,77091 | 812,2853637 | 12 |
|  | MDP24 | 270,713993 | 217,7028878 | 12 |
|  | MDP48 | 239,19515 | 176,6199815 | 12 |
| **TNF-α** | Basal prod | 11,2354373 | 11,50886464 | 12 |
|  | LPS24 | 266,192243 | 181,0378213 | 12 |
|  | LPS48 | 119,80522 | 116,5273247 | 12 |
|  | MDP24 | 110,741981 | 74,40010453 | 12 |
|  | MDP48 | 45,5127201 | 39,26743959 | 12 |
| **IL-10** | Basal prod | 5,49077523 | 4,907114569 | 12 |
|  | LPS24 | 196,661182 | 90,87530525 | 12 |
|  | LPS48 | 159,900187 | 132,3201141 | 12 |
|  | MDP24 | 101,076498 | 77,68346244 | 12 |
|  | MDP48 | 76,441085 | 78,05878068 | 12 |
| **IL-17A** | Basal prod | 2,31315251 | 0,887030747 | 12 |
|  | LPS24 | 2,67575814 | 1,166520534 | 12 |
|  | LPS48 | 2,4721102 | 0,563569852 | 12 |
|  | MDP24 | 2,61411674 | 1,266671448 | 12 |
|  | MDP48 | 3,18244653 | 1,962197583 | 12 |
| **IL-1β** | ΔLPS24 | 909,493142 | 497,1819208 | - |
|  | ΔLPS48 | 1136,93621 | 808,487919 | - |
|  | ΔMDP24 | 200,627159 | 169,9819984 | - |
|  | ΔMDP48 | 196,360447 | 149,9548119 | - |
| **TNF-α** | ΔLPS24 | 251,693402 | 170,634077 | - |
|  | ΔLPS48 | 113,252857 | 114,2525033 | - |
|  | ΔMDP24 | 112,339997 | 75,24474268 | - |
|  | ΔMDP48 | 38,9603562 | 37,45023716 | - |
| **IL-10** | ΔLPS24 | 206,650057 | 98,14614934 | - |
|  | ΔLPS48 | 163,64035 | 135,7090607 | - |
|  | ΔMDP24 | 85,6941811 | 69,2165285 | - |
|  | ΔMDP48 | 72,4400712 | 77,37882433 | - |
| **TNF-α** | ΔLPS24 | 0,13979211 | 0,839177703 | - |
|  | ΔLPS48 | 0,25184137 | 0,723752787 | - |
|  | ΔMDP24 | 0,02729906 | 1,118752954 | - |
|  | ΔMDP48 | 0,96217769 | 1,407152485 | - |

**Supplementary Table 6.** Row data used in statistical analysis (Z-scores) for Index case 1.

| ***Cytokine*** | ***Condition*** | ***Mean*** | ***SD*** | ***N*** |
| --- | --- | --- | --- | --- |
| **IL-1β** | Basal prod | 306,3331186 | 0 | 1 |
|  | LPS24 | 2654,098511 | 0 | 1 |
|  | LPS48 | 3390,841935 | 0 | 1 |
|  | MDP24 | 2223,790276 | 0 | 1 |
|  | MDP48 | 2489,875741 | 0 | 1 |
| **TNF-α** | Basal prod | 61,58145801 | 0 | 1 |
|  | LPS24 | 716,5272004 | 0 | 1 |
|  | LPS48 | 410,2863286 | 0 | 1 |
|  | MDP24 | 669,6237416 | 0 | 1 |
|  | MDP48 | 413,1016397 | 0 | 1 |
| **IL-10** | Basal prod | 294,35673 | 0 | 1 |
|  | LPS24 | 813,334829 | 0 | 1 |
|  | LPS48 | 890,6417847 | 0 | 1 |
|  | MDP24 | 776,7607433 | 0 | 1 |
|  | MDP48 | 870,5474144 | 0 | 1 |
| **IL-17A** | Basal prod | 11,7584903 | 0 | 1 |
|  | LPS24 | 14,31693713 | 0 | 1 |
|  | LPS48 | 13,66969898 | 0 | 1 |
|  | MDP24 | 15,63997054 | 0 | 1 |
|  | MDP48 | 10,96262405 | 0 | 1 |
| **IL-1β** | ΔLPS24 | 2347,765392 | 0 | - |
|  | ΔLPS48 | 3084,508816 | 0 | - |
|  | ΔMDP24 | 1917,457157 | 0 | - |
|  | ΔMDP48 | 2183,542622 | 0 | - |
| **TNF-α** | ΔLPS24 | 654,9457424 | 0 | - |
|  | ΔLPS48 | 348,7048705 | 0 | - |
|  | ΔMDP24 | 608,0422836 | 0 | - |
|  | ΔMDP48 | 351,5201817 | 0 | - |
| **IL-10** | ΔLPS24 | 518,978099 | 0 | - |
|  | ΔLPS48 | 596,2850547 | 0 | - |
|  | ΔMDP24 | 482,4040133 | 0 | - |
|  | ΔMDP48 | 576,1906844 | 0 | - |
| **TNF-α** | ΔLPS24 | 2,558446834 | 0 | - |
|  | ΔLPS48 | 1,911208688 | 0 | - |
|  | ΔMDP24 | 3,881480245 | 0 | - |
|  | ΔMDP48 | -0,795866242 | 0 | - |

**Supplementary Table 7.** Row data used in statistical analysis (Z-scores) for Sibling of patient 1.

| ***Cytokine*** | ***Condition*** | ***Mean*** | ***SD*** | ***N*** |
| --- | --- | --- | --- | --- |
| **IL-1β** | Basal prod | 88,57478657 | 0 | 1 |
|  | LPS24 | 1747,620575 | 0 | 1 |
|  | LPS48 | 2132,62543 | 0 | 1 |
|  | MDP24 | 1175,403388 | 0 | 1 |
|  | MDP48 | 716,3701048 | 0 | 1 |
| **TNF-α** | Basal prod | 21,05434853 | 0 | 1 |
|  | LPS24 | 1084,300929 | 0 | 1 |
|  | LPS48 | 344,1159501 | 0 | 1 |
|  | MDP24 | 592,5878009 | 0 | 1 |
|  | MDP48 | 168,4760353 | 0 | 1 |
| **IL-10** | Basal prod | 58,65487757 | 0 | 1 |
|  | LPS24 | 1039,928224 | 0 | 1 |
|  | LPS48 | 841,2113999 | 0 | 1 |
|  | MDP24 | 768,5727386 | 0 | 1 |
|  | MDP48 | 550,5500963 | 0 | 1 |
| **IL-17A** | Basal prod | 5,828779522 | 0 | 1 |
|  | LPS24 | 12,44920447 | 0 | 1 |
|  | LPS48 | 10,47458233 | 0 | 1 |
|  | MDP24 | 10,48615252 | 0 | 1 |
|  | MDP48 | 7,995275702 | 0 | 1 |
| **IL-1β** | ΔLPS24 | 1659,045788 | 0 | - |
|  | ΔLPS48 | 2044,050643 | 0 | - |
|  | ΔMDP24 | 1086,828601 | 0 | - |
|  | ΔMDP48 | 627,7953182 | 0 | - |
| **TNF-α** | ΔLPS24 | 1063,246581 | 0 | - |
|  | ΔLPS48 | 323,0616016 | 0 | - |
|  | ΔMDP24 | 571,5334524 | 0 | - |
|  | ΔMDP48 | 147,4216868 | 0 | - |
| **IL-10** | ΔLPS24 | 981,273346 | 0 | - |
|  | ΔLPS48 | 782,5565223 | 0 | - |
|  | ΔMDP24 | 709,9178611 | 0 | - |
|  | ΔMDP48 | 491,8952187 | 0 | - |
| **TNF-α** | ΔLPS24 | 6,620424952 | 0 | - |
|  | ΔLPS48 | 4,645802804 | 0 | - |
|  | ΔMDP24 | 4,657372994 | 0 | - |
|  | ΔMDP48 | 2,16649618 | 0 | - |

**Supplementary Table 8.** Row data used in statistical analysis (Z-scores) for Mother of patient 1.

| ***Cytokine*** | ***Condition*** | ***Mean*** | ***SD*** | ***N*** |
| --- | --- | --- | --- | --- |
| **IL-1β** | Basal prod | 110,07 | 0 | 1 |
|  | LPS24 | 2113,413328 | 0 | 1 |
|  | LPS48 | 2190,696146 | 0 | 1 |
|  | MDP24 | 1018,427565 | 0 | 1 |
|  | MDP48 | 1008,791539 | 0 | 1 |
| **TNF-α** | Basal prod | 45,22474158 | 0 | 1 |
|  | LPS24 | 1212,681198 | 0 | 1 |
|  | LPS48 | 1000,774688 | 0 | 1 |
|  | MDP24 | 707,3502328 | 0 | 1 |
|  | MDP48 | 550,7253045 | 0 | 1 |
| **IL-10** | Basal prod | 37,46790187 | 0 | 1 |
|  | LPS24 | 161,7227544 | 0 | 1 |
|  | LPS48 | 204,6756628 | 0 | 1 |
|  | MDP24 | 138,0676408 | 0 | 1 |
|  | MDP48 | 154,9031375 | 0 | 1 |
| **IL-17A** | Basal prod | 8,280730305 | 0 | 1 |
|  | LPS24 | 10,95215222 | 0 | 1 |
|  | LPS48 | 9,282742484 | 0 | 1 |
|  | MDP24 | 9,838117422 | 0 | 1 |
|  | MDP48 | 8,409846722 | 0 | 1 |
| **IL-1β** | ΔLPS24 | 2003,343328 | 0 | - |
|  | ΔLPS48 | 2080,626146 | 0 | - |
|  | ΔMDP24 | 908,357565 | 0 | - |
|  | ΔMDP48 | 898,721539 | 0 | - |
| **TNF-α** | ΔLPS24 | 1167,456457 | 0 | - |
|  | ΔLPS48 | 955,549946 | 0 | - |
|  | ΔMDP24 | 662,1254912 | 0 | - |
|  | ΔMDP48 | 505,500563 | 0 | - |
| **IL-10** | ΔLPS24 | 124,2548525 | 0 | - |
|  | ΔLPS48 | 167,2077609 | 0 | - |
|  | ΔMDP24 | 100,5997389 | 0 | - |
|  | ΔMDP48 | 117,4352356 | 0 | - |
| **TNF-α** | ΔLPS24 | 2,671421916 | 0 | - |
|  | ΔLPS48 | 1,00201218 | 0 | - |
|  | ΔMDP24 | 1,557387117 | 0 | - |
|  | ΔMDP48 | 0,129116417 | 0 | - |

**Supplementary Table 9.** Row data used in statistical analysis (Z-scores) for Father of patient 1.

| ***Cytokine*** | ***Condition*** | ***Mean*** | ***SD*** | ***N*** |
| --- | --- | --- | --- | --- |
| **IL-1β** | Basal prod | 78,91463719 | 0 | 1 |
|  | LPS24 | 616,5573703 | 0 | 1 |
|  | LPS48 | 999,5873824 | 0 | 1 |
|  | MDP24 | 428,8140739 | 0 | 1 |
|  | MDP48 | 429,8773832 | 0 | 1 |
| **TNF-α** | Basal prod | 16,29964152 | 0 | 1 |
|  | LPS24 | 367,0732589 | 0 | 1 |
|  | LPS48 | 107,3800515 | 0 | 1 |
|  | MDP24 | 170,8866308 | 0 | 1 |
|  | MDP48 | 88,79214178 | 0 | 1 |
| **IL-10** | Basal prod | 71,36129083 | 0 | 1 |
|  | LPS24 | 134,9769852 | 0 | 1 |
|  | LPS48 | 555,4940003 | 0 | 1 |
|  | MDP24 | 472,450484 | 0 | 1 |
|  | MDP48 | 399,3428047 | 0 | 1 |
| **IL-17A** | Basal prod | 7,509271315 | 0 | 1 |
|  | LPS24 | 9,09199812 | 0 | 1 |
|  | LPS48 | 9,676380135 | 0 | 1 |
|  | MDP24 | 10,80125068 | 0 | 1 |
|  | MDP48 | 9,504292411 | 0 | 1 |
| **IL-1β** | ΔLPS24 | 537,6427331 | 0 | - |
|  | ΔLPS48 | 920,6727452 | 0 | - |
|  | ΔMDP24 | 349,8994367 | 0 | - |
|  | ΔMDP48 | 350,962746 | 0 | - |
| **TNF-α** | ΔLPS24 | 350,7736174 | 0 | - |
|  | ΔLPS48 | 91,08040994 | 0 | - |
|  | ΔMDP24 | 154,5869893 | 0 | - |
|  | ΔMDP48 | 72,49250025 | 0 | - |
| **IL-10** | ΔLPS24 | 63,61569438 | 0 | - |
|  | ΔLPS48 | 484,1327095 | 0 | - |
|  | ΔMDP24 | 401,0891931 | 0 | - |
|  | ΔMDP48 | 327,9815138 | 0 | - |
| **TNF-α** | ΔLPS24 | 1,582726805 | 0 | - |
|  | ΔLPS48 | 2,16710882 | 0 | - |
|  | ΔMDP24 | 3,291979369 | 0 | - |
|  | ΔMDP48 | 1,995021096 | 0 | - |

**Supplementary Table 10.** Row data used in statistical analysis (Z-scores) for Index case 2.

| ***Cytokine*** | ***Condition*** | ***Mean*** | ***SD*** | ***N*** |
| --- | --- | --- | --- | --- |
| **IL-1β** | Basal prod | 22,41791578 | 0 | 1 |
|  | LPS24 | 2456,029492 | 0 | 1 |
|  | LPS48 | 2223,172121 | 0 | 1 |
|  | MDP24 | 208,2418176 | 0 | 1 |
|  | MDP48 | 186,0593244 | 0 | 1 |
| **TNF-α** | Basal prod | 31,18538455 | 0 | 1 |
|  | LPS24 | 1107,451128 | 0 | 1 |
|  | LPS48 | 531,2107055 | 0 | 1 |
|  | MDP24 | 213,6583211 | 0 | 1 |
|  | MDP48 | 150,5619944 | 0 | 1 |
| **IL-10** | Basal prod | 3,280128696 | 0 | 1 |
|  | LPS24 | 73,91666281 | 0 | 1 |
|  | LPS48 | 36,3786892 | 0 | 1 |
|  | MDP24 | 37,91418225 | 0 | 1 |
|  | MDP48 | 26,55948228 | 0 | 1 |
| **IL-17A** | Basal prod | 2,71142137 | 0 | 1 |
|  | LPS24 | 8,206392598 | 0 | 1 |
|  | LPS48 | 7,302205759 | 0 | 1 |
|  | MDP24 | 8,933238335 | 0 | 1 |
|  | MDP48 | 6,605084476 | 0 | 1 |
| **IL-1β** | ΔLPS24 | 2433,611576 | 0 | - |
|  | ΔLPS48 | 2200,754205 | 0 | - |
|  | ΔMDP24 | 185,8239018 | 0 | - |
|  | ΔMDP48 | 163,6414086 | 0 | - |
| **TNF-α** | ΔLPS24 | 1076,265744 | 0 | - |
|  | ΔLPS48 | 500,0253209 | 0 | - |
|  | ΔMDP24 | 182,4729365 | 0 | - |
|  | ΔMDP48 | 119,3766098 | 0 | - |
| **IL-10** | ΔLPS24 | 70,63653411 | 0 | - |
|  | ΔLPS48 | 33,0985605 | 0 | - |
|  | ΔMDP24 | 34,63405356 | 0 | - |
|  | ΔMDP48 | 23,27935359 | 0 | - |
| **TNF-α** | ΔLPS24 | 5,494971228 | 0 | - |
|  | ΔLPS48 | 4,590784389 | 0 | - |
|  | ΔMDP24 | 6,221816965 | 0 | - |
|  | ΔMDP48 | 3,893663106 | 0 | - |
